# SIM2s directed Parkin-mediated mitophagy promotes mammary epithelial cell differentiation

**DOI:** 10.1038/s41418-023-01146-9

**Published:** 2023-03-25

**Authors:** Lilia Sanchez, Jessica Epps, Steven Wall, Cole McQueen, Scott J. Pearson, Kelly Scribner, Elizabeth A. Wellberg, Erin D. Giles, Monique Rijnkels, Weston W. Porter

**Affiliations:** 1 Department of Veterinary Physiology and Pharmacology; College of Veterinary Medicine, Texas A&M University, College Station, TX 77843 USA; 2 Department of Toxicology, CTEH, 5120 Northshore Drive, Little Rock, AR 72118 USA; 3 Stephenson Cancer Center, University of Oklahoma Health Sciences Center, Oklahoma City, OK 73104 USA; 4 School of Kinesiology, University of Michigan, 830 N University Ave, Ann Arbor, MI 48109 USA; 5 Department of Veterinary Integrative Biosciences; College of Veterinary Medicine, Texas A&M University, College Station, TX 77843 USA

**Keywords:** Autophagy, Metabolic disorders

## Abstract

The functionally differentiated mammary gland adapts to extreme levels of stress from increased demand for energy by activating specific protective mechanisms to support neonatal health. Here, we identify the breast tumor suppressor gene, single-minded 2 s (SIM2s) as a novel regulator of mitophagy, a key component of this stress response. Using tissue-specific mouse models, we found that loss of *Sim2* reduced lactation performance, whereas gain (overexpression) of *Sim2s* enhanced and extended lactation performance and survival of mammary epithelial cells (MECs). Using an in vitro model of MEC differentiation, we observed SIM2s is required for Parkin-mediated mitophagy, which we have previously shown as necessary for functional differentiation. Mechanistically, SIM2s localizes to mitochondria to directly mediate Parkin mitochondrial loading. Together, our data suggest that SIM2s regulates the rapid recycling of mitochondria via mitophagy, enhancing the function and survival of differentiated MECs.

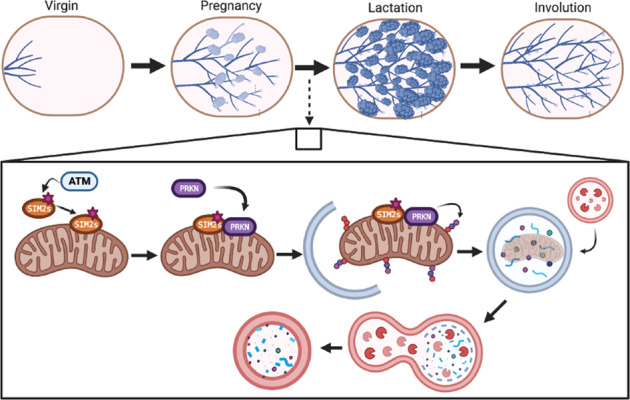

## Introduction

In response to compromised homeostasis, cells adapt or die, and this decision is guided by the origin, severity, and duration of the stress as well as by the stress response and the cellular context. Insults to cellular homeostasis include DNA damage, nutrient deprivation, xenobiotics exposure, hypoxia, and energy demand, which activate stress responses to counteract cellular damage and preserve survival. These responses are vital to tissue health and development. For example, mammary epithelial cells (MECs) sustain cell survival to undergo differentiation under the severe stress of energy and nutrient demand to support the production of milk, which is essential for neonatal growth [1, 2]. To adapt to this demand, MECs upregulate cell stress responses [3–6]. These stress responses protect lactating MECs for a finite period, which can be determined if lactation is artificially extended [3, 7]. We have previously demonstrated that overexpression (~3-fold) of single-minded 2 s (*Sim2s*) specifically in the murine mammary gland prolongs the survival of MECs beyond forced involution, enhances the expression of differentiation-dependent genes in lactating MEC, and promotes a gene expression profile in the virgin gland characteristic of differentiated MEC, suggesting that SIM2s contributes to the establishment and maintenance of a differentiated MEC state [8]. Furthermore, we have established that SIM2s is required for mammary ductal development; however, the regulatory signaling and downstream mechanisms remain unclear [8–10]. Here, we demonstrate that SIM2s enhances MEC survival and function by engaging mitophagy, a key component of many stress responses [11].

Mitophagy refers to the autophagic clearance of mitochondria, an essential component of mitochondrial and cellular homeostasis. Highly metabolic tissues such as the heart maintain steady-state levels of mitophagy, but this process also occurs in response to internal or external stress stimuli [12]. There are two major mitophagy mechanisms: ubiquitin-mediated and receptor-mediated [13]. Ubiquitin-mediated mitophagy is canonically triggered by mitochondrial depolarization, which occurs when mitochondrial membrane potential collapses [14, 15]. Receptor-mediated mitophagy can be triggered by hypoxia or nutrient starvation, which cause recruitment of mitophagy receptors to damaged mitochondria [16, 17]. Both mechanisms function to remove damaged or dysfunctional mitochondria via the autophagy machinery. Here, we demonstrate that SIM2s interacts with mitophagy machinery to induce MECs differentiation and adapt to the energy demand of lactation by promoting the rapid recycling of mitochondria.

SIM2s is a member of the bHLH-PAS family of transcription factors, which classically function as environmental sensors [18]. bHLH-PAS factors can bind DNA, proteins, and small molecules, allowing them to respond to numerous environmental stimuli and enact transcriptional responses [19, 20]. Although many environmental stimuli, such as oxygen tension and dioxin, have been described for other bHLH-PAS proteins, very few stimuli have been reported for SIM2s. We have recently shown that DNA damage stimulates the phosphorylation of SIM2s in an ATM-dependent manner, suggesting that SIM2s indirectly senses DNA damage and contributes to the DNA damage response [21, 22]. Based on this, we hypothesized that SIM2s may operate as an environmental sensor to detect lactogenic differentiation cues and respond to the energetic stress experienced by differentiated MECs.

We have previously shown autophagy regulates functional differentiation of MEC. To address the function of SIM2s in the lactogenic differentiation of MECs, we employed mammary gland-specific mouse models of *Sim2s* loss and overexpression (gain) and evaluated their lactation performance. Pairing these models with models of in vitro MEC differentiation, we demonstrate that SIM2s contributes to the lactogenic differentiation of MECs by interacting with and enhancing mitophagy. In doing so, SIM2s supports MEC survival and adaptation to the energy demand of lactation by driving the rapid turnover of mitochondria. We propose that SIM2s is a novel mitophagy factor in MECs. This work provides important insight into how cells adapt to energy stress during differentiation and has implications for both tissue development and disease.

## Results

### Loss of *Sim2* impairs mammary gland function in vivo

Proper mammary gland development and differentiation of MECs is essential to mammalian lactation, and dysregulation of these processes can result in dysfunction and disease with serious implications for the individual and its offspring. Our group has previously established that *Sim2* is required for normal ductal development in a mouse mammary bud transplant model [9], and several groups have shown that complete loss of *Sim2* results in perinatal lethality in mice [23–25]. Because neither of these models can be used to study the effect of loss of *Sim2* on mammary gland development and MEC differentiation through gestation and lactation, we engineered tissue-specific conditional knockout mice via a floxed *Sim2* allele (*Sim2*^*fl/fl*^) (Supplementary Fig. 1). *Cre* recombinase expression was driven with the whey acidic protein (*Wap*) promoter, which is expressed in MECs from mid-pregnancy through lactation [26]. We show loss of *Sim2s* expression in mammary glands of *Sim2*^*fl/fl*^ mice at mid-lactation (day 13) compared to control mice at mid-lactation (day 10), via immunohistochemistry staining of mammary gland tissue at mid lactation (Supplementary Fig.1), confirming our earlier findings [22]. Of note, the short splice variant of *Sim2*, *Sim2s*, is the only detectable variant in the murine mammary gland [9, 27, 28], and loss of *Sim2* is considered equivalent to loss of *Sim2s* in this model.

**Fig. 1:**
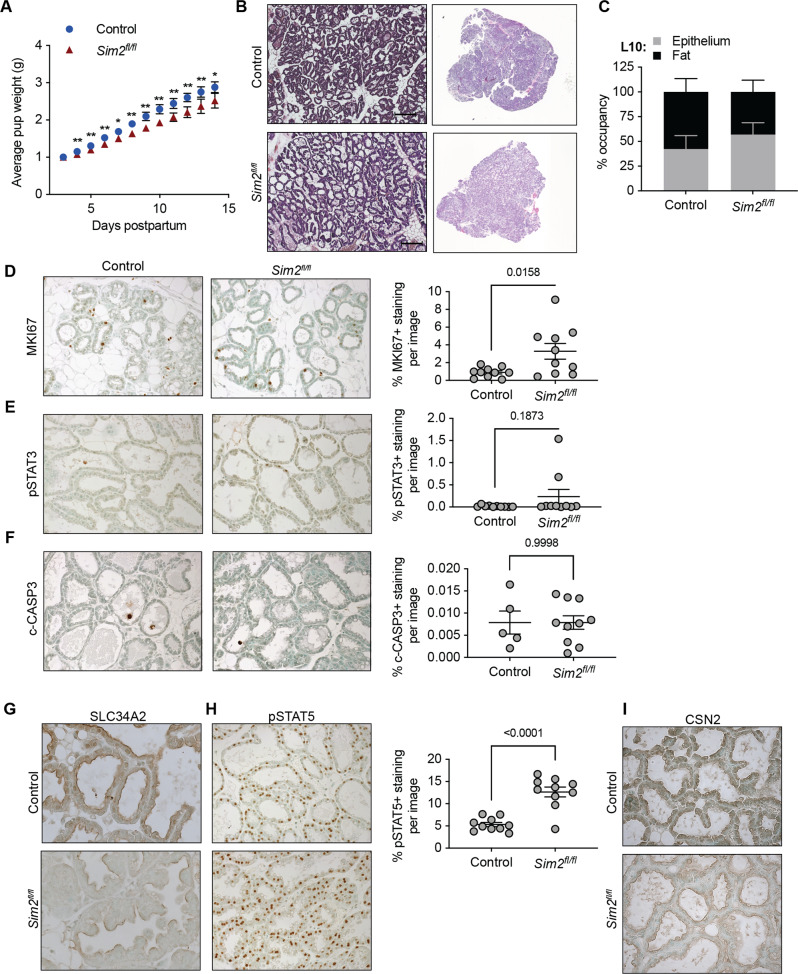
Loss of *Sim2* impairs lactation performance in mice. **A** Average weight of cross-fostered pups nursed by control and *Sim2*^*fl/fl*^ mice (*n* = 5 control, *n* = 4 *Sim2*^*fl/fl*^). **B** Representative H&E-stained mammary sections at lactation day 10. **C** Quantitation of epithelial content and fat pad occupancy in slide scans of H&E stained mammary gland sections (*n* = 5 control, *n* = 4 *Sim2*^*fl/fl*^). **D** Immunostaining for MKI67 at lactation day 2 and quantitation of positive staining (*n* = 2 mice per group). Immunostaining at lactation day 10 and quantitation of positive staining for pSTAT3 (**E**) and c-CASP3 (**F**) (*n* = 2 mice per group). **G** Immunostaining for SLC34A2 at lactation day 10. **H** Immunostaining for pSTAT5 at lactation day 10 and quantitation of positive staining (*n* = 2 mice per group). **I** Immunostaining for CSN2 at lactation day 10. Error bars indicate the mean ± SD. *p < 0.05, **p < 0.01, scale bars: 100 μm.

To address whether loss of *Sim2* influenced mammary gland function, we compared lactation performance by measuring the weight gain of pups nursed by control and *Sim2*^*fl/fl*^ dams. To remove bias originating from the genotype of the pups, we cross-fostered the first litters of control and *Sim2*^*fl/fl*^ mice with ten weight-normalized wild type pups per dam. To account for slight differences (±1 day) in the timing of cross-fostering, we compared weight gain relative to the first day of cross-fostering. We found that by day 4 of lactation, pups nursed by *Sim2*^*fl/fl*^ dams weighed significantly less than those nursed by control dams (Fig. 1A), and this deficiency persisted through the end of lactation. The reduced weight of pups nursed by *Sim2*^*fl/fl*^ mice suggests that loss of *Sim2* negatively impacts lactation and MEC function.

To identify the underlying drivers of reduced lactation performance in *Sim2*^*fl/fl*^ mice, we evaluated mammary gland architecture by hematoxylin and eosin (H&E) staining in control and *Sim2*^*fl/fl*^ mice. We did not observe gross differences in gland structure (Fig. 1B) or epithelial content (Fig. 1C) at peak lactation (day 10). These results suggest that mammary-specific loss of *Sim2* in pregnancy and lactation does not impair MEC proliferation or alveolar expansion. To confirm that proliferation was not impaired by loss of *Sim2* earlier in lactation, we examined the expression of marker of proliferation Ki-67 (MKI67, also known as Ki-67) at lactation day 2. We found an increase in *Sim2*^*fl/fl*^ mice compared to controls (Fig. 1D), suggesting that reduced lactation was not driven by impaired proliferation in *Sim2*^*fl/f*^ mice. Furthermore, we did not observe any differences in the expression of involution (phosphorylated STAT3; signal transducer and activator of transcription 3) [29, 30] or cell death (c-CASP3; cleaved-caspase 3) markers at lactation day 10 (Fig. 1E, F). These findings indicate that the reduced weight of pups nursed by *Sim2*^*fl/fl*^ dams cannot be accounted for by reduced proliferation, pre-mature involution, or cell death, which would all alter total MEC number.

Because the difference in pup weight could not be explained by a difference in MEC number, we evaluated MEC function by analyzing markers of differentiation. During differentiation, MECs form alveolar units that express nutrient transporters on their apical membrane to secrete nutrients into the alveolar lumen [31, 32]. Thus, the polarization of these transporters to the apical membrane can be used as a marker of MEC differentiation. Consistent with development of polarized alveolar units, we found that SLC34A2 (solute carrier family 34 member 2) was expressed on the apical membrane of MECs in both control and *Sim2*^*fl/fl*^ mice by lactation day 10 (Fig. 1G). STAT5 (signal transducer and activator of transcription 5) is a master transcriptional regulator of milk protein genes, including beta-casein (CSN2) [33, 34], and is activated by phosphorylation (pSTAT5) [35, 36]. We observed robust activation of STAT5 by lactation day 10 in the mammary glands of both control and *Sim2*^*fl/fl*^ mice (Fig. 1H). Although we observed significantly enhanced STAT5 activation in *Sim2*^*fl/fl*^ mice, we found that CSN2 expression was mildly suppressed in *Sim2*^*fl/fl*^ mice (Fig. 1I). These results suggest that loss of *Sim2* does not negatively impact the activation of STAT5 or the development of alveolar units; however, the reduction in CSN2 expression suggests that SIM2 may influence differentiation by affecting CSN2 expression independent of STAT5 transcriptional regulation.

### SIM2s promotes mammary gland function in vivo

**Fig. 2:**
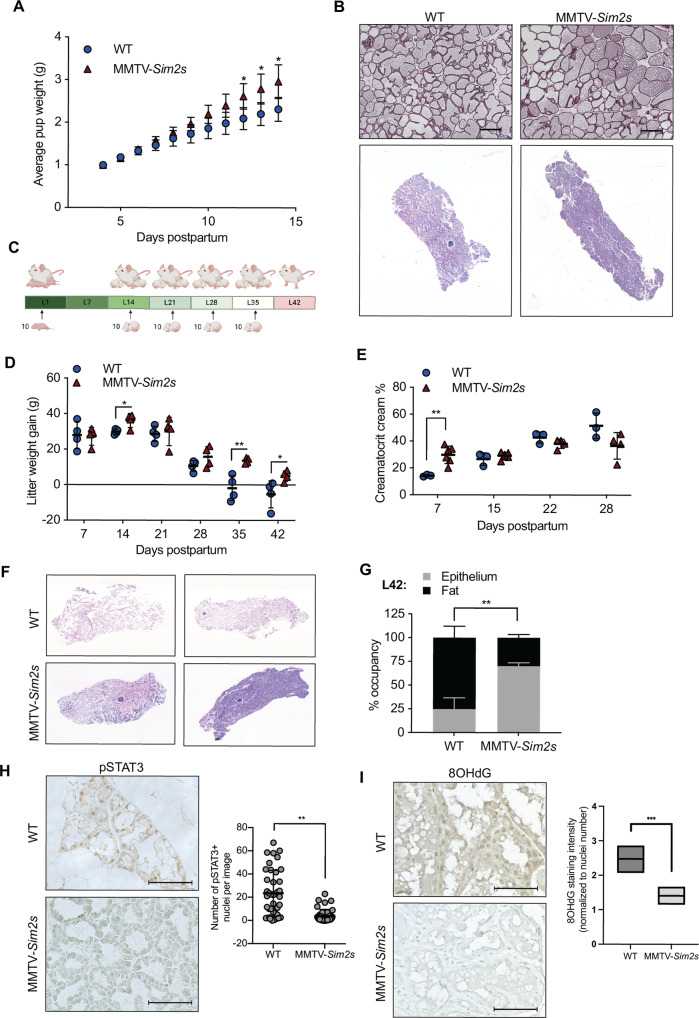
Moderate overexpression of *Sim2s* extends lactation performance. **A** Cross-fostered pups were weighed daily to assess weight gain when nursed by WT and MMTV-*Sim2s* mice (*n* = 3 WT, *n* = 7 MMTV-*Sim2s*). **B** Representative H&E staining in mouse mammary gland sections at lactation day 10. **C** Experimental design for extended lactation studies. **D** Weekly weight gain of cross-fostered litters nursed by WT and MMTV-*Sim2s* mice during extended lactation study (*n* = 4 mice per group). **E** Crude milk fat percentage of milk collected from WT and MMTV-*Sim2s* mice during extended lactation study (*n* = 3 WT, *n* = 6 MMTV-*Sim2s*, milk could not be collected from every mouse at every timepoint). **F** Whole slide scans of H&E-stained mammary gland sections at day 42 of lactation. **G** Quantitation of epithelial and fat pad occupancy from whole slide scan H&E images (*n* = 4 mice per group). **H** Immunostaining for pSTAT3 in WT and MMTV-*Sim2s* mice mammary gland tissue at lactation day 42, and quantitation of pSTAT3-positive nuclei per image. Ten images per mouse were assessed (*n* = 2 mice per group). **I** Immunostaining for 8-OHdG at lactation day 28 in WT and MMTV-*Sim2s* mammary sections with quantification of 8-OHdG staining intensity normalized to number of nuclei. Error bars indicate the mean ± SD. *p < 0.05, **p < 0.01, scale bars: 100 μm.

In contrast to the effects of *Sim2* loss, we have previously shown that developmentally-relevant (~3-fold) overexpression of *Sim2s*, driven by the mouse mammary tumor virus (MMTV), promotes precocious MEC differentiation, delays involution, and maintains alveolar structures after the cessation of lactation in the mouse mammary gland [8, 10]. These studies suggest that overexpression of *Sim2s* promotes a differentiated phenotype and maintains MEC survival. To functionally confirm these findings, we performed cross-fostering experiments and compared the weight gain of pups nursed by wild type (WT) or MMTV-*Sim2s* dams. We found that pups nursed by MMTV-*Sim2s* dams gained significantly more weight by day 12 of lactation compared to pups nursed by WT dams (Fig. 2A). Consistent with our previous report, we did not observe differences in the glands of these mice by H&E staining (Fig. 2B). These results suggest that overexpression of *Sim2s* confers a functional advantage in terms of lactation performance, but the underlying source of this advantage remains unclear.

To further evaluate whether *Sim2s* overexpression prolongs MEC survival and function, we performed an extended lactation study using WT and MMTV-*Sim2s* mice. Lactation was prolonged to 42 days by repeated cross-fostering (Fig. 2C). Initial litters were cross-fostered with 10, weight-matched, 1-day-old pups. Beginning on day 14, litters were replaced with 10, weight-matched, 7-day-old pups every week to maintain suckling stimulus and milk let down.^3^ Litter weights were recorded every week, and pup weight gain was calculated for each 7-day period. We found that MMTV-*Sim2s* dams were able to sustain significantly higher litter weight gain for two weeks (days 35 and 42) beyond normal weaning (day 21) (Fig. 2D). Analysis of the cream percentage from the milk of WT and MMTV-*Sim2s* mice indicated that increased milk fat content may contribute to an early weight gain advantage but could not fully account for the prolonged lactation ability of MMTV-*Sim2s* mice (Fig. 2E).

Based on the lactation performance advantage of MMTV-*Sim2s* dams and our previous work showing delayed involution in MMTV-*Sim2s* mice, we anticipated that MEC survival would be enhanced in the mammary glands of MMTV-*Sim2s* mice after extended lactation. Indeed, we found that MMTV-*Sim2s* glands contained significantly more epithelial content by day 42 of lactation compared to WT glands (Fig. 2F, G). Moreover, MMTV-*Sim2s* glands contained significantly fewer pSTAT3 positive nuclei on day 42, suggesting that canonical involution signaling was delayed in these glands compared to WT glands (Fig. 2H). Possible sources of oxidative damage by reactive oxygen species, especially during a stressful process such lactation, could be through a variety of causes. Hadsell et al. [3] suggested that, especially during a prolonged lactation cycle, mammary epithelial cells undergo dynamics that replicated an accelerated aging model. Their study proposes mitochondrial protein suffers increased oxidative damage at the onset of lactation and again with prolonged lactation, along with an increase in apoptosis. As a specific marker of oxidative DNA damage, 8-OHdG is used as a measure of oxidative stress [7, 37]. We found reduced levels of 8-OHdG in the cytoplasm of MECs from MMTV-*Sim2s* mice at lactation day 28 compared to WT mice (Fig. 2I). These data suggest that reduced oxidative stress in MMTV-*Sim2s* MECs may contribute to the observed survival advantage.

Altogether, these results suggest that mammary-specific overexpression of *Sim2s* enhances and prolongs the function of MECs and delays involution. It is important to note that the loss of *Sim2* did not result in pre-mature involution or MEC death in *Sim2*^*fl/fl*^ mice, suggesting that many factors contribute to the maintenance of MEC function and that SIM2s enhances and supports this function.

### SIM2s promotes an energetic phenotype and alters mitochondrial homeostasis

**Fig. 3:**
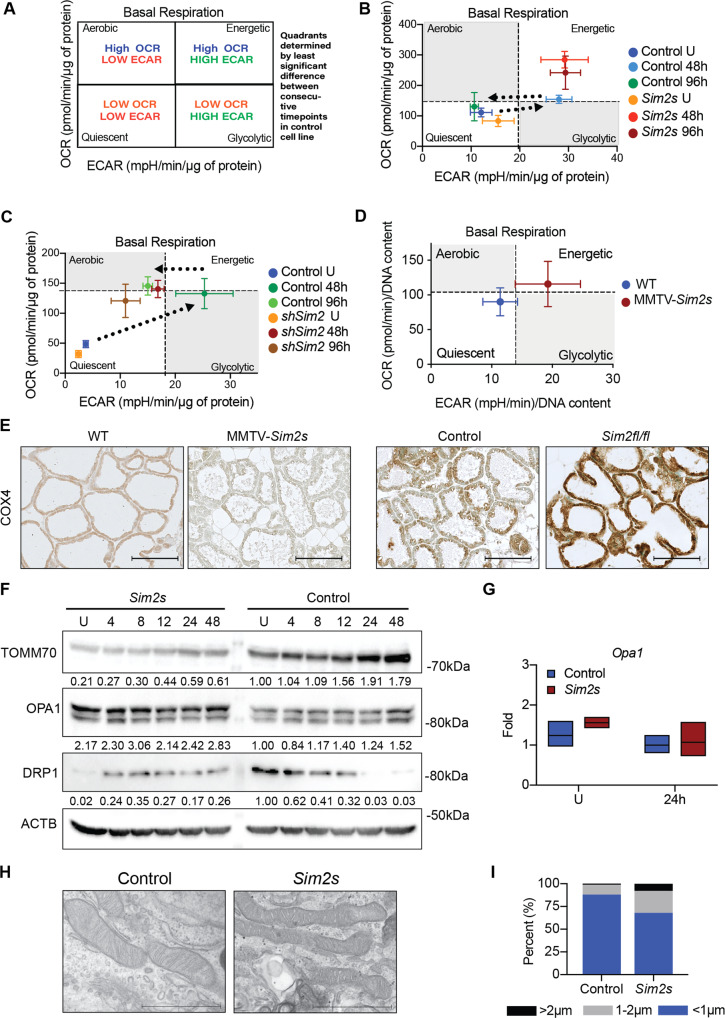
SIM2s influences the energetic phenotype and mitochondrial homeostasis of MECs. **A** Energy phenotype model. ECAR extracellular acidification rate, OCR oxygen consumption rate. **B** Basal energy phenotype of control and *Sim2s* overexpressing HC11 cells at an undifferentiated state as well as at 48 and 96 h of differentiation. (*n* = 3 independent experiments with ≥6 technical replicates each). **C** Basal energy phenotype of *sh*Control and *shSim2* HC11 cells at an undifferentiated state as well as at 48 and 96 h of differentiation (*n* = 2 independent experiments with ≥10 technical replicates each). Superimposed dotted arrows indicate the control cell trend across differentiation. **D** Basal energy phenotype of primary MECs isolated from virgin control or MMTV-*Sim2s* mice and subjected to hormonal induction for 24 h. (*n* = 3 independent experiments with ≥11 technical replicates each). **E** Immunostaining for mitochondrial marker COX4 in WT and MMTV-*Sim2s* mice as well as control and *Sim2*^*fl/fl*^ mice at lactation day 10 (*n* = 2 mice per group). **F** Protein levels of mitochondrial homeostasis markers in control and *Sim2s* overexpressing HC11 cells across differentiation. **G** *Opa1* expression in undifferentiated and 24 h differentiated control and *Sim2s* HC11 cells (*n* = 3 independent experiments with biological triplicates and technical duplicates). **H** TEM images of mitochondria in control and *Sim2s* HC11 cells at 24 h of differentiation. **I** Quantification of mitochondrial lengths from TEM images. Lengths were divided into three categories corresponding to punctate/round mitochondria (<1 μm), mid-length mitochondria (1–2 μm), and elongated mitochondria (>2 μm) and presented as percentages of total mitochondria. Error bars indicate the mean ± SD. Scale bars: 100 μm in **E** and 1 μm in **H**.

To better understand how SIM2s supports and enhances MEC function, we overexpressed and knocked down *Sim2s* in the HC11 mouse mammary epithelial cell line, as previously described [10]. We then investigated the impact of *Sim2s* on the energy phenotype of HC11 cells. We have previously established that the energy phenotype is an important functional aspect and marker of HC11 cell differentiation [38]. The energy phenotype is a comparison of the relative balance between mitochondrial respiration and glycolysis, which were simultaneously measured as the oxygen consumption rate (OCR) and the extracellular acidification rate (ECAR), respectively [39, 40]. These measurements were then paired as x and y coordinates to assign treatment groups to one of four relative energy phenotypes: quiescent, glycolytic, aerobic, or energetic (Fig. 3A) [40]. The cell is quiescent when it is not primarily using either glycolysis or mitochondrial respiration as a main energetic pathway. Glycolytic means the cell is primarily using glycolysis, while aerobic means the cell is primarily using mitochondrial respiration. Finally, the cell would be classified as energetic if the cell is employing both metabolic pathways.

Using this method, we asked whether *Sim2s* affected the basal energy phenotype of differentiated MECs. We have previously established that HC11 cells attain an energetic phenotype at peak differentiation (~48 h) [38]. Interestingly, we found that HC11-*Sim2s* cells were substantially more energetic than control cells (Fig. 3B), and this advantage persisted out to 96 h of differentiation at a level that was not different from that of 48 h. This finding suggests that *Sim2s* enhances and prolongs an energetic phenotype, which is consistent with the functional advantages observed in our lactation studies. In contrast, we found that stable knock-down of *Sim2* in HC11 cells did not alter mitochondrial respiration at peak differentiation but did prevent *shSim2* cells from attaining a fully energetic phenotype (Fig. 3C). Based on our previous work, these findings suggest that *Sim2s* enhances and maintains the differentiation of HC11 cells.

To validate these findings, we isolated primary MECs (PMECs) from WT and MMTV-*Sim2s* mice and maintained them in culture to perform energy phenotyping. As isolation of lactating PMECs is challenging due to milk and lipid content, we isolated PMECs from nulliparous females and cultured them with lactogenic hormones for 24 h prior to analysis. We found that PMECs from MMTV-*Sim2s* mice demonstrated a more energetic phenotype than PMECs from WT mice (Fig. 3D). These results support our in vitro observations and suggest that *Sim2s* also promotes an energetic phenotype during MEC differentiation ex vivo.

Based on the dramatic differences in energy phenotypes with gain and loss of *Sim2s*, we sought to determine whether total mitochondrial number was affected by *Sim2s*. Indeed, we observed opposing trends in immunostaining for the inner mitochondrial membrane protein COX4 (cytochrome c oxidase subunit 4) in our transgenic models. COX4 immunostaining demonstrated a slight reduction with overexpression of *Sim2s* and was substantially elevated with loss of *Sim2* (Fig. 3E). We hypothesized that this contrast in mitochondrial accumulation between MMTV-*Sim2s* and *Sim2*^*fl/fl*^ mammary glands may be due to alterations in mitochondrial homeostasis, which is maintained through the coordination of mitochondrial fusion, fission, biogenesis, and mitophagy [41, 42].

To evaluate mitochondrial homeostasis in our transgenic models, we immunostained mammary sections from lactation day 10 for optic atrophy 1 (OPA1, fusion), dynamin 1 related (DRP1, fission), PPARG coactivator 1 alpha (PPARGC1A, biogenesis), and sequestosome 1 (SQSTM1, mitophagy) (Supplementary Fig. 2). Interestingly, PPARGC1A was elevated in both MMTV-*Sim2s* and *Sim2*^*fl/fl*^ mice compared to WT and control mice, respectively, despite clear differences in total mitochondrial accumulation. OPA1 did not appear to be differentially expressed between any of the genotypes. DRP1 and SQSTM1 also did not vary substantially between WT and MMTV-*Sim2s* mice; however, we noted several alterations between control and *Sim2*^*fl/fl*^ mice. For example, DRP1 and SQSTM1 staining observed around lipid droplets at the apical membrane in control, WT, and MMTV-*Sim2s* mice appeared to be disrupted in *Sim2*^*fl/fl*^ mice (Supplementary Fig. 2). Altogether, these results suggest that mitochondrial homeostasis may be disrupted in *Sim2*^*fl/fl*^ mice but do not clarify the differences in mitochondrial accumulation or energetic phenotypes observed in MMTV-*Sim2s* mice.

As we were interested in the survival and functional advantages conferred by overexpression of *Sim2s*, we performed our mechanistic evaluation in HC11-*Sim2s* and control cells. We began by assessing mitochondrial homeostasis during HC11 cell differentiation in more detail. In control HC11 cells, we found that TOMM70 (translocase of outer mitochondrial membrane 70) and OPA1 expression increased up to peak differentiation, and DRP1 expression decreased across the same time (Fig. 3F). Interestingly, overexpression of *Sim2s* altered this expression profile by reducing the levels of TOMM70 and by deregulating the pattern of OPA1 and DRP1 expression. TOMM70 was used as a surrogate for total mitochondrial number, and the observed downregulation of TOMM70 with overexpression of *Sim2s* was consistent with the reduced levels of COX4 observed in MMTV-*Sim2s* mice. Because OPA1 levels appeared to be elevated with *Sim2s* overexpression, we examined *Opa1* mRNA levels as well as the physical morphology of mitochondria from *Sim2s* cells. We found that *Sim2s* overexpression did not alter *Opa1* mRNA levels, suggesting that *Opa1* is not a transcriptional target of SIM2s (Fig. 3G). Further, analysis of mitochondrial length from transmission electron microscopy (TEM) images revealed that HC11-*Sim2s* cells contained a higher percentage of elongated mitochondria by 24 h of differentiation compared to control cells (Fig. 3H, I), suggesting that *Sim2s* impacts mitochondrial fusion as well as total mitochondrial number. Of note, the underlying cause of reduced mitochondrial accumulation with *Sim2s* overexpression in MMTV-*Sim2s* mice and HC11-*Sim2s* cells remains unclear.

### Mitophagy is altered by SIM2s

**Fig. 4:**
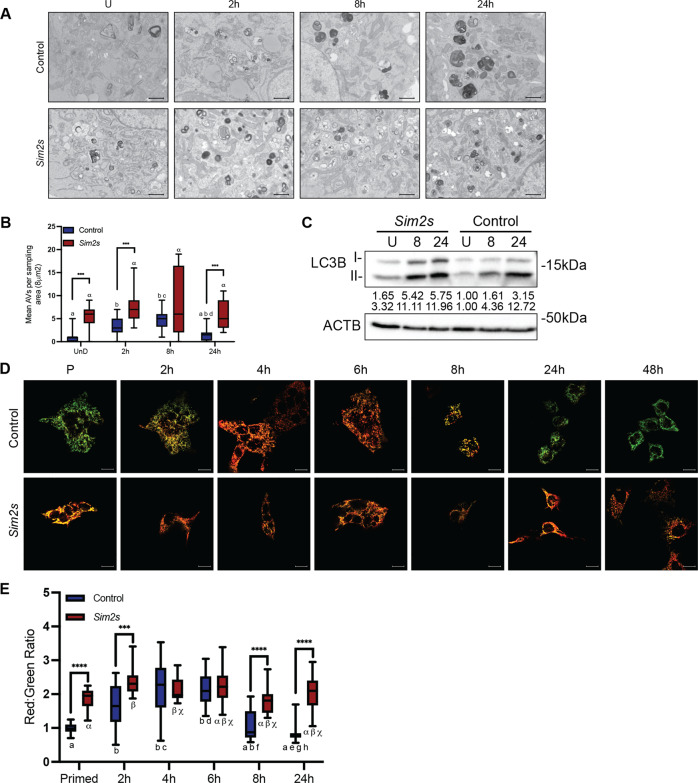
*Sim2s* enhances mitophagy in vitro. **A** TEM images of control and *Sim2s* overexpressing HC11 cells across differentiation. Hours indicate differentiation time points. **B** Quantification of mean autophagic vesicles per sampling area (approximately 8 μm^2^). **C** Protein levels of MAP1LC3B I and II in control and *Sim2s* HC11 cells across differentiation. **D** MitoTimer images of live control and *Sim2s* HC11 cells across differentiation. **E** Quantification of the red to green fluorescent ratio from the MitoTimer images in **D** (*n* = 10 images per time point per cell line, representative of three independent experiments). Box and whisker graphs center around the mean, and whiskers extend to the minimum and maximum values. U undifferentiated, P primed 24 h.**p < 0.01, For all variables with the same letter, the difference between the means is not statistically significant; scale bars: 1  μm in **A** and 100 μm in **D**.

Our analyses thus far imply that overexpression of *Sim2s* enhances and prolongs the function and energy phenotype of MECs and corresponds with reduced mitochondrial accumulation and increased mitochondrial fusion and biogenesis. As mitophagy functions to reduce mitochondrial number and maintain the health of the mitochondrial population, we hypothesized that mitophagy may be enhanced with *Sim2s* overexpression. Despite being increasingly recognized as a major player in tissue development and differentiation, mitophagy remains difficult to quantify, and no single marker can be used to assess its prevalence. Therefore, we first examined the effect of *Sim2s* overexpression on autophagic flux during HC11 cell differentiation using TEM (Fig. 4A). We observed dramatic accumulation of autophagic vesicles in HC11-*Sim2s* cells compared to control cells at every timepoint. The autophagic vesicles in control cells demonstrated progressive maturation across differentiation, whereas *Sim2s* cells contained numerous and diverse autophagic vesicles (Fig. 4B). Moreover, TEM evaluation of the CIT3 mouse mammary epithelial cell line also demonstrated enhanced accumulation of diverse autophagic vesicles with *Sim2s* overexpression (Supplementary Fig. 3). We confirmed the enhanced presence of autophagic membranes in HC11-*Sim2s* cells by evaluating the protein expression of microtubule-associated protein 1 light chain 3B (MAP1LC3B, also known as LC3B), which is a standard method of monitoring autophagy and phagophore formation [43]. We again observed induction of autophagy during differentiation in control cells and enhancement with *Sim2s* (Fig. 4C). These results suggest that autophagy is enhanced in HC11-*Sim2s* cells and support the additional evaluation of mitophagy.

To evaluate mitophagy, we employed the *MitoTimer* fluorescent reporter system [44–46]. The *pMitoTimer* construct, driven by the constitutive CMV promoter, localizes to mitochondria via a cytochrome c oxidase subunit 8 (COX8) targeting sequence, fluoresces green when newly synthesized, and shifts irreversibly to red once oxidized [44]. High ratios of red to green fluorescence intensity indicate increased mitochondrial protein oxidation and suggest elevated levels of mitophagy. Using this system, control and *Sim2s* HC11 cells transiently expressing *pMitoTimer* were monitored by live cell imaging over the course of differentiation (Fig. 4D). Ratiometric analysis revealed that HC11-*Sim2s* cells exhibited higher mitochondrial protein oxidation, which was maintained throughout differentiation (Fig. 4E). Control HC11 cells demonstrated a progressive oxidation pattern that is similar to and validates our previous findings in WT HC11 cells, suggesting that mitophagy is engaged to facilitate MEC differentiation. These results are also consistent with observations in CIT3 cells showing decreased levels of MitoTracker and increased levels of LysoTracker at peak differentiation in *Sim2s* cells (Supplementary Fig. 3). Of note, mitochondria have been reported to elongate in periods of elevated autophagy to escape mitophagic degradation [47]. Thus, it remains unclear whether the mitochondrial elongation observed in HC11-*Sim2s* cells is mediated directly by overexpression of *Sim2s* or indirectly in response to the observed autophagic environment. Although accumulation of autophagic vesicles and elevated *pMitoTimer* ratios could indicate impaired mitochondrial turnover, the lack of cell death and the enhanced differentiation observed with *Sim2s* overexpression suggest that *Sim2s* promotes MEC function and survival by enhancing mitophagy.

### SIM2s is activated by ATM in response to hormonal cues

**Fig. 5:**
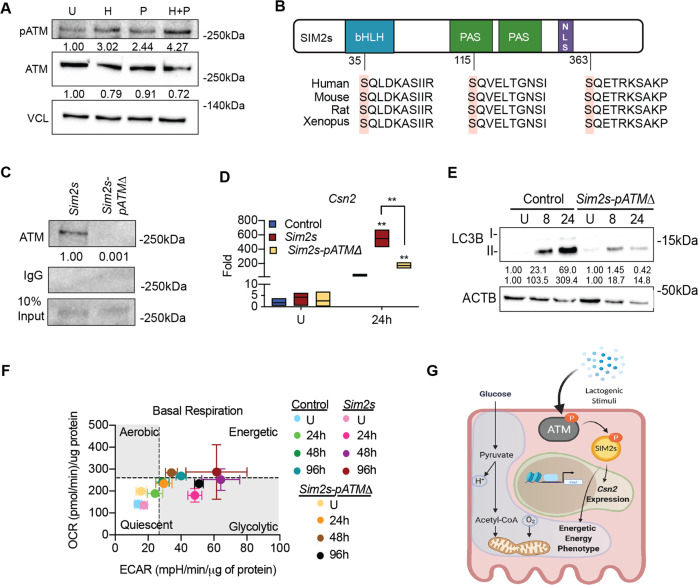
Hormone-mediated ATM activation impacts SIM2s function. **A** Expression of activated (phosphorylated) ATM in HC11 cells at an undifferentiated state and 24 h after adding hydrocortisone alone, prolactin alone, or hydrocortisone + prolactin. **B** ATM consensus phosphorylation sites on SIM2s. **C** Co-immunoprecipitation of ATM and FLAG in *Sim2s* or *Sim2s*-*pATMΔ* HC11 cells at 24 h differentiation. **D** mRNA expression of differentiation-dependent *Csn2* in control, *Sim2s*, and *Sim2s*-*pATMΔ* HC11 cell lines (*n* = 2 independent experiments with biological triplicates and technical duplicates). **E** Protein levels of MAP1LC3B I and II in control and *Sim2s*-*pATMΔ* HC11 across differentiation. **F** Basal energy phenotype in control, *Sim2s*, and *Sim2s*-*pATMΔ* HC11 cells across differentiation (*n* = 2 independent experiments with ≥10 technical replicates each). **G** Proposed model of ATM-mediated phosphorylation of SIM2s and its downstream effects. bHLH basic helix-loop-helix, ECAR extracellular acidification rate, H hydrocortisone, NLS nuclear localization sequence, OCR oxygen consumption rate, P prolactin, PAS PER-ARNT-SIM, U undifferentiated. Error bars indicate the mean ± SD. **p < 0.01.

There is increasing evidence that the protein kinase ATM (ataxia telangiectasia mutated) plays critical roles in response to mitochondrial dysfunction independent of its DNA damage response activity [48]. Intriguingly, ATM is also required for mammary gland functional differentiation [49, 50], and conditional deletion of *Atm* in the mouse mammary gland results in lactation failure [51]. This is significant because we recently demonstrated that ATM binds and phosphorylates SIM2s in response to replication stress [21]. As individual loss of either ATM or SIM2s negatively impacts lactation, we hypothesized that ATM and SIM2s may cooperate to sense differentiation cues. Indeed, we found that ATM was abundantly activated after the addition of both hydrocortisone and prolactin during HC11 cell differentiation (Fig. 5A). As a positive control, we treated undifferentiated HC11 cells with hydroxyurea for 24 h to induce DNA damage response and activate ATM (Supplementary Fig. 5). We have also previously shown that mutation of three ATM consensus sites on SIM2s (Fig. 5B) impairs ATM-mediated phosphorylation of SIM2s and downstream SIM2s function [21]. Indeed, we found that ATM interacted with FLAG-tagged SIM2s at 24 h of differentiation in HC11 cells, but this interaction was lost if point mutations at the three ATM consensus sites were introduced into the *Sim2s* (*Sim2s-pATMΔ*) expression vector (Fig. 5C). To assess downstream SIM2s function in differentiation, we compared the induction of *Csn2* at 24 h in HC11 cells overexpressing *Sim2s* or *Sim2s-pATMΔ*. We found that *Csn2* expression was significantly elevated with overexpression of *Sim2s*, as we have previously reported [10]; however, overexpression of the mutated form of *Sim2s* did not enhance expression of *Csn2* to the same extent (Fig. 5D). Similarly, *Sim2s-pATMΔ* cells expressed decreased autophagy induction compared to control cells (Fig. 5E) and did not reach or maintain the same energy phenotype as *Sim2s* cells (Fig. 5F). Together, these results suggest that ATM-mediated phosphorylation of SIM2s may serve as an upstream environmental sensor of differentiation (Fig. 5G).

### Localization of SIM2s during MEC differentiation

**Fig. 6:**
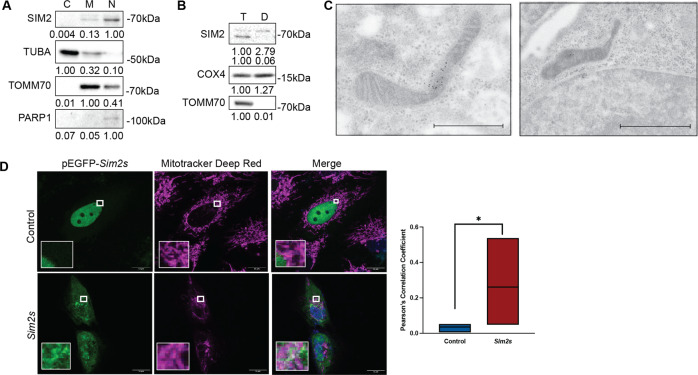
SIM2s localizes to mitochondria. **A** Cytoplasmic, mitochondrial, and nuclear cell fractions in 24 h differentiated HC11 cells showing protein levels of SIM2, TUBA, TOMM70, and PARP1 in each fraction. **B** Immunogold staining for SIM2 in 24 h differentiated HC11 cells. Black particles indicate gold labeling of SIM2 in mitochondria and nuclei. **C** Total and digested mitochondrial fractions in 24 h differentiated HC11 cells showing SIM2, COX4, and TOMM70 protein levels. **D** Representative live-cell images of control and SUM159-*Sim2s* cells transiently transfected with pEGFP*-Sim2s* and stained with Mitotracker DeepRed with quantification by Pearson’s correlation coefficient. C cytoplasmic, D digested, M mitochondrial, N nuclear. T total, D Digested. Scale bars in **C**: 1 μm. Error bars indicate the mean *p < 0.05.

After sensing an environmental cue, bHLH-PAS proteins typically translocate to the nucleus to engage a transcriptional response [20]. Alternatively, many non-transcriptional functions have also been described for bHLH-PAS proteins, including AHR and HIF1A [52, 53]. Therefore, we sought to determine the localization of SIM2s in differentiated HC11 cells. We collected cytosolic, mitochondrial, and nuclear fractions from 24 h differentiated HC11 cells and found that SIM2s was strongly localized in the nuclear fraction, as expected. Unexpectedly, we found that SIM2s was also present in the mitochondrial fraction (Fig. 6A). The relative purity of the fractions was assessed by the expression of cytosolic (alpha-tubulin; TUBA), mitochondrial (TOMM70), and nuclear (poly[ADP-ribose] polymerase 1; PARP1) proteins. To determine if SIM2s was imported into mitochondria, we subjected half of a mitochondrial preparation to proteinase K digestion to degrade outer mitochondrial membrane (OMM) proteins and left the other half intact. Digestion of OMM proteins was confirmed by the presence or absence of the OMM protein TOMM70 or the inner mitochondrial membrane protein COX4. Although TOMM70 was efficiently degraded in the digested (D) fraction, COX4 and SIM2 were present in both total (T) and digested fractions (Fig. 6B). The mitochondrial localization of SIM2s was confirmed by immunogold in 24 h differentiated HC11 cells, which clearly showed the presence of gold nanoparticles in both mitochondria and nuclei (Fig. 6C). To further verify SIM2s mitochondrial localization, SUM159 breast cancer cells were used to better visualize physical interactions, because they do not express basal levels of *SIM2s* [54, 55]. We transiently transfected SUM159 control and SUM159-*Sim2s* cells with a SIM2s labeled EGFP vector (pEGFP-SIM2s) and co-stained with Mitotracker Deep Red. Pearson’s correlation coefficient showed significantly higher colocalization of SIM2s and mitochondria in SUM159-*Sim2s* cells (Fig. 6D). Furthermore, line-scanning confocal analysis of undifferentiated transfected HC11 cells showed nuclear localization of SIM2s. However, at 24 h differentiation, we observed increased cytoplasmic localization of pEGFP-SIM2s and Mitotracker Deep Red, providing additional evidence of SIM2s localization to the mitochondria during differentiation (Supplementary Fig. 4).

**Fig. 7:**
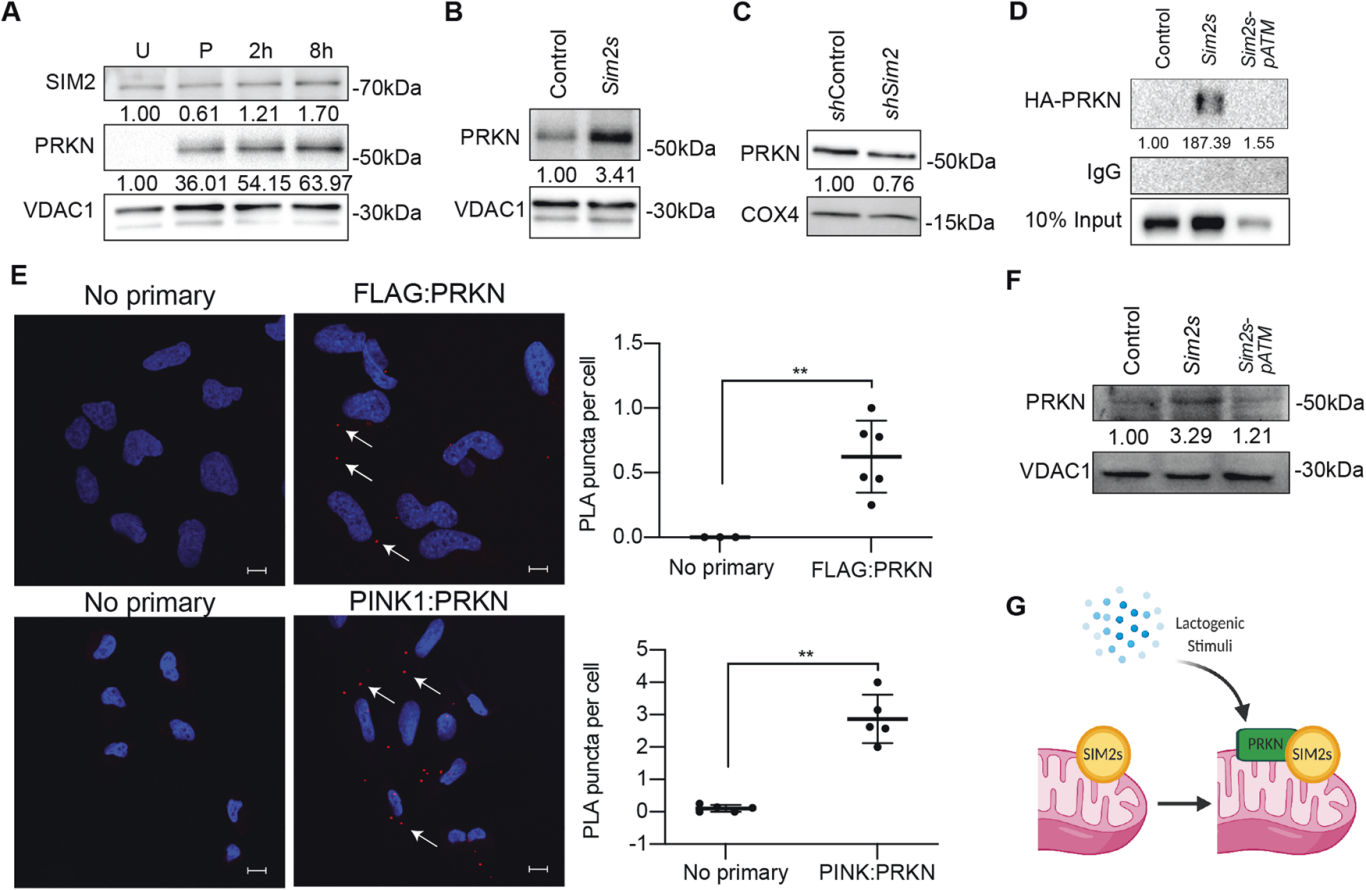
SIM2s interacts with and regulates Parkin mitochondrial loading. **A** Mitochondrial fractions showing accumulation of SIM2, PRKN, and VDAC1 protein levels across HC11 cell differentiation. VDAC1 was used as a protein loading control, all differentiated timepoints were normalized to the undifferentiated timepoint. **B** Mitochondrial fractions from control and *Sim2s* HC11 cells at 24 h of differentiation with protein levels of PRKN and VDAC1. **C** Mitochondrial fractions from *sh*Control and *shSim2* HC11 cells at 24 h of differentiation showing protein levels of PRKN and COX4. **D** Co-immunoprecipitation of HA-PRKN and FLAG in control, *Sim2s*, or *Sim2s*-*pATMΔ* SUM159 cells. **E** Interaction of FLAG (tagged to SIM2s) and PRKN visualized by PLA in SUM159 cells. Interactions were detected as a fluorescent red dot and quantified as puncti per cell. PINK1:PRKN interactions were used as a positive assay control (*n* = 3–6 images per cell line per antibody, representative of two independent experiments). **F** Mitochondrial fractions from control, *Sim2s*, and *Sim2s*-*pATMΔ* HC11 cells at 24 h of differentiation with protein levels of PRKN and VDAC1. **G** Model of SIM2-PRKN interaction in response to hormonal stimuli; P primed for 24 h, PLA proximity ligation assay, U undifferentiated. Error bars indicate the mean ± SD. **p < 0.01. Scale bars: 10 μm in **E**.

We have previously shown that the mitophagy factor PRKN (parkin RBR E3 ubiquitin protein ligase) is critical for HC11 cell differentiation [38], and, interestingly, SIM2 has been shown to directly interact with PRKN [56]. Although the SIM2:PRKN interaction was postulated to occur in the nucleus, no supportive evidence for the localization of the interaction was provided [56]. Our findings suggest that SIM2s and PRKN may instead interact at the mitochondria. To investigate the relationship between SIM2s and PRKN, we isolated mitochondrial fractions at different timepoints during HC11 cell differentiation and evaluated the presence of SIM2 and PRKN. We found that SIM2 was present at every timepoint, whereas PRKN only localized to mitochondria during and after priming (Fig. 7A). To determine if the level of PRKN varied with *Sim2s* expression, we evaluated PRKN levels in mitochondrial fractions from HC11-*Sim2s* and HC11-*shSim2* cell lines. Interestingly, mitochondrial fractions from HC11-*Sim2s* cells showed a substantially elevated PRKN level compared to control cells at 24 h of differentiation (Fig. 7B). In contrast, PRKN expression was mildly decreased in mitochondrial fractions from *shSim2* cells compared to *sh*Control cells (Fig. 7C). Finally, we evaluated the physical interaction of SIM2s and PRKN by co-immunoprecipitation and proximity ligation assay (PLA) in SUM159-*Sim2s* breast cancer cells. We observed a dramatic increase of SIM2s and HA-PRKN interaction in SUM159-*Sim2s* cells compared to control cells. This interaction was lost in SUM159- *Sim2s-pATMΔ* cells (Fig. 7D). We also found that PRKN interacted with both FLAG-tagged SIM2s and its canonical mitophagy partner PINK1 (PTEN induced kinase 1) The PINK1:PRKN interaction was included as a positive control. In a proximity ligation assay two antibodies raised in different species (rabbit and mouse) are used to recognize two proteins of interest. The antibodies are conjugated to a pair of oligonucleotides (PLUS and MINUS probes). When these probes come into proximity with each other (about 40 nm) connector oligos will connect the PLA probes and are ligated to form closed circular DNA template. A polymerase and fluorescently labeled oligonucleotides (red fluorescent label) are added, which results in rolling circle amplification, and the signal is detected as a red fluorescent dot. These dots or puncti can then be quantified and can demonstrate interactions between distinct proteins, in our case either PINK1:PRKN, or FLAG-Sim2s:PRKN. (Fig. 7E). Finally, mitochondrial fractions from HC11-*Sim2s*-*pATMΔ* cells showed a decrease in PRKN loading compared to control cells and HC11-*Sim2s*. (Fig. 7F). Together, these results suggest that SIM2s may modulate mitochondrial homeostasis and MEC function through non-canonical and non-transcriptional mechanisms (Fig. 7G).

## Discussion

The increased metabolic demand for protein, carbohydrate, and lipid production brought on by lactogenic differentiation necessitates cellular mechanisms that mitigate stress and damage. We suggest that this stress may be mitigated differently in MECs during two key developmental time frames: the initiation of lactogenic differentiation and the maintenance of a highly productive differentiated state. We have previously shown that the mitochondrial network is remodeled and programmed mitophagy is required for the initiation of lactogenic differentiation [38]. We hypothesized that a remodeled mitochondrial network was required to induce differentiation and meet the energetic demand of lactation and that these highly productive mitochondrial networks were prone to exhaustion and production of reactive oxidants. Our strategy was to identify factors that enhanced the differentiation and function and survival of MECs in this environment and to investigate mechanisms that mitigate stress during lactation. Our contrasting models of tissue-specific *Sim2s* expression in the mouse mammary gland allowed us to modify the functional and energetic status of MECs in vivo and identified mitophagy as a potential mechanism of stress mitigation. It is evident from these studies that alterations in mitophagy and mitochondrial function impact the functional differentiation of MECs.

Mitochondrial function is essential to energy production and the function of metabolically active tissues, including the mammary gland; however, mitochondrial homeostasis is relatively understudied in the mammary epithelium. A key facet of mitochondrial homeostasis is the selective elimination of mitochondria, which is accomplished through two pathways: ubiquitin-mediated mitophagy and receptor-mediated mitophagy. Ubiquitin-mediated mitophagy occurs primarily through the PINK1/PRKN pathway and is initiated by the depolarization of mitochondrial membrane potential and the stabilization of PINK1 on the OMM [14, 15]. PINK1 then recruits PRKN to ubiquitinate OMM proteins. These ubiquitin chains are phosphorylated by PINK1 and recognized by autophagy receptors, such as OPTN and SQSTM1, which are typically conjugated to LC3 on the phagophore membrane [17]. Receptor-mediated mitophagy is more direct in that it involves the recognition of LC3 on the phagophore membrane by mitochondrially localized proteins that contain an LIR motif [17]. Although these mechanisms are generally accepted, many studies have shown that mitophagy pathways are highly tissue-specific, and unique mitophagy factors have been identified in many tissues [17]. We recently demonstrated that PRKN is a key factor in the survival of differentiating MECs and accumulates in mitochondrial fractions only after the initiation of differentiation [38]. Interestingly, we found that PRKN localization was not dependent on mitochondrial depolarization, suggesting that PRKN-mediated mitophagy occurs independent of PINK1 in MECs [38]. This hypothesis is supported by observations that basal mitophagy in highly metabolic tissues is not impacted by the loss of *Pink1* and is likely to involve tissue-specific factors [12]. In this study, we show that SIM2s enhances mitophagy by regulating recruitment of PRKN to mitochondria in differentiated MECs. We further showed that this functional interaction is dependent on phosphorylation of SIM2s by ATM. These results suggest the interesting hypothesis that SIM2s functions as a novel mitophagy receptor to maintain mitochondrial homeostasis in differentiated MECs upon phosphorylation by lactogenic-induction and activation of ATM.

ATM, known for its role in DNA damage, has also been implicated in maintaining mitochondrial homeostasis [48, 57, 58]. For example, loss of ATM in ataxia-telangiectasia patients impairs mitochondrial function and mitophagy independent of its DNA damage response [58]. Here, we show that ATM is activated during MEC differentiation and interacts with SIM2s. This observation suggests that ATM contributes to mitochondrial homeostasis, possibly by recognizing lactogenic stimuli and phosphorylating mitophagy substrates, such as SIM2s. We have previously shown that SIM2s is phosphorylated by ATM in response to DNA damage in breast cancer cell lines [21], and others have reported that ATM is activated by oxidative stress [48]. As both DNA damage and oxidative stress are likely to occur during lactation due to the strained environment of MECs, we cannot exclude the possibility that ATM is activated by alternative mechanisms during lactation. Regardless of the activation stimulus of ATM, it is clear from our study that ATM-mediated phosphorylation of SIM2s contributes to MEC differentiation and may be a mechanism by which ATM regulates mitophagy.

The use of contrasting *Sim2s* transgenic models allowed us to modulate lactation performance in vivo and examine the molecular outcomes. Although we noted reduced lactation performance in *Sim2*^*fl/fl*^ mice, we did not find major differences in MEC proliferation, alveolar expansion, or apoptosis, suggesting that the inherent function of MECs was negatively impacted by loss of *Sim2*. We did observe subtle alterations in the expression patterns of mitochondrial homeostasis factors around lipid droplets in *Sim2*^*fl/fl*^ mice compared to all other genotypes. The presence of SQSTM1 and DRP1 around lipid droplets in the lactating mammary gland was surprising and was disrupted in *Sim2*^*fl/fl*^ mice. This observation could suggest that mitochondrial fission and mitophagy contribute to the secretion of lipids during lactation; however, we did not observe a significant difference in the total lipid percentage of milk isolated from control and *Sim2*^*fl/fl*^ mice (Supplementary Fig. 1). Additional studies are underway to further define the molecular cause of reduced lactation performance in *Sim2*^*fl/fl*^ mice, including the relationship between the mitochondria and other organelles. There is increasing evidence of the importance of proper inter-organelle communication when mitigating cellular stress. Preliminary analysis of electron microscopy images of control and *Sim2*^*fl/fl*^ mammary gland tissue revealed subtle changes in ER-mitochondria contact sites. Future studies will investigate the role these physical interactions may play in the molecular processes underlying functional mammary gland differentiation.

Here, we show that SIM2s enhances the differentiation, function, and survival of MECs in part by regulating mitophagy through the interaction of SIM2s and PRKN. We suggest that mitophagy supports the survival of differentiated MECs by mitigating the damage caused by dysfunctional or exhausted mitochondria in a metabolically demanding environment. We anticipate that these findings will open the door for additional study of how SIM2s and mitochondrial homeostasis contribute to cell fate and differentiation decisions in both normal and disease states.

## STAR methods

### Animals

All mice were cared for in accordance with the Texas A&M University ethical guidelines and Animal Care and Use Committee. Mice were provided access to food and water ad libitum and were housed under standard 12 h photoperiods. As this study concerned mammary gland development, only female mice were used. No method of randomization was used. Three to five female mice were analyzed for each timepoint. Timepoints were chosen as representatives of the morphological and functional changes that occur during mammary gland development. After sacrifice, mammary glands were harvested for whole mounting, immunostaining, and nucleic acid extraction.

Transgenic mice were bred and genotyped by the Texas A&M Institute for Genomic Medicine. MMTV-*Sim2s* mice have been described previously and were bred in an FVB background [10]. *Sim2*^*fl/fl*^ mice were developed using a cre-loxP recombinase strategy in a mixed 129/SvEv and C57BL/6 background as follows (Supplementary Fig. 1). The targeting vector was designed to span the genomic DNA region 2473 bp upstream and 3770 bp downstream of *Sim2* exon 1 and contained loxP sites on either side of exon 1. The targeting vector was electroporated into 129/SvEv embryonic stem cells. Stem cells containing the transgene were then injected into C57BL/6 albino blastocysts, and chimeras were bred using C57BL/6 N females to obtain the target germline. To excise the *Neo* cassette, heterozygous mice were crossed with ACTB:FLPe mice [(B6.Cg-Tg(ACTFLPe)9205Dym/J) (Jax Stock 005703)] and produced *Sim2*^*fl/+*^ mice. To excise the loxP-flanked region of *Sim2* and visualize recombinase activity, *Sim2*^*fl/+*^ mice were crossed with *Wap*-cre [(B6.Cg-Tg(Wap-cre)11738Mam/JKnwJ; Jax Stock 008735)] and *mTmG* [(B6.129(Cg)-Gt(ROSA)26Sor^tm4(ACTB-tdTomato,-EGFP)luo^/J; Jax stock 007676)] mice [59]. *Wap*-cre mice express cre recombinase under control of the whey acidic protein (*Wap*) promoter and were selected because *Wap* is expressed specifically in MECs shortly before and during lactation. We also included the genetic reporter *mTmG* to distinguish recombined and non-recombined cells in our model. Using this model, non-recombinant cells express tdTomato, and recombinant cells express EGFP, which should indicate excision of *Sim2* exon 1. Final genotypes were generated by crossing *Sim2*^*fl/+*^*;Wap*^*Cre/+*^ mice to *Sim2*^*fl/+*^*;mTmG/mTmG* mice to produce *Sim2*^*fl/fl*^*;Wap*^*Cre/+*^*;mTmG* mice (*Sim2*^*fl/fl*^) and littermate *Wap*^*Cre/+*^*;mTmG* (control) mice. PCR genotyping was performed with the following primers: F4-GCAGATTCGAGTGAGAGACT, F2-AAGAGGACTGGAGGGAGAGG, and R2-ACCCCAGACCTGATTCACTG. The PCR reaction mix consisted of Platinum Taq DNA Polymerase (Invitrogen), 10x PCR buffer (Invitrogen), 50 mM MgCl_2_, and 10 mM dNTP mix. Reactions conditions were as follows: initial denaturation for 2 min at 94 °C and 30 sec at 95 °C; followed by 36 cycles of 10 sec denaturation at 95 °C, 30 sec annealing at 60 °C, and 1 min elongation at 65 °C; and concluded with 5 min at 65 °C. PCR products were run on 2% agarose gels to visualize resulting bands. F4 and R2 primers give two products corresponding to the WT (319 bp) and floxed (564 bp) alleles. F2 and R2 primers give only the deleted flox (402 bp) allele (Supplementary Fig. 1).

Cross-fostering studies were performed by replacing the first litter of transgenic mice with 10, weight-matched ICR pups to remove bias stemming from pup genotype. Litter weights were recorded every morning, and pup weight gain was calculated relative to the first day of cross-fostering to account for differences (±1 day) in timing of cross-fostering and variation in the weight of ICR litters. Lactation was extended by additional rounds of cross-fostering on lactation days 14, 21, 28, and 35. Litter weight gain for extended lactation studies was calculated by subtracting the litter weight at the end of each seven-day period from the litter weight at the beginning of the seven day period.

### Milk collection and creamatocrit

Milk samples were collected at lactation days 7, 15, 22, and 28. Milk collection was performed as previously described [3, 7, 60]. Briefly, dams were separated from pups for a minimum of 2 h prior to milk collection. Mice were anesthetized under 2–5% isoflurane, and nipple areas were disinfected with 70% ethanol. An intramuscular injection of 0.5i.u. oxytocin was administered in each rear leg to stimulate milk release. Milk was collected by vacuum-assisted suction from each nipple, and mice were allowed to fully recover from anesthesia prior to reintroduction to their litter. Milk was aliquoted and frozen for additional analyses, and creamatocrit analysis was performed without freezing and within an hour of collection. Creamatocrit analysis was performed by gently inverting milk samples and loading 10 μL in a Drummond microcapillary haematocrit tube (VWR) [61, 62]. Tubes were sealed on one end with Hemato-seal (Thermo Fisher Scientific) and centrifuged at 1500 × *g* at room temperature to separate the cream layer. The linear length of the cream component was divided by the total length of the milk sample to determine the cream percentage of each sample.

### Immunostaining

Tissue samples were fixed overnight at 4 °C in 4% paraformaldehyde and stored in 70% ethanol at 4 °C until processing. Samples were processed, sectioned, and H&E stained by the Texas A&M University College of Veterinary Medicine & Biomedical Science Histology Laboratory. Immunostaining was performed as previously described [10, 38]. Briefly, tissue sections were de-waxed and rehydrated in graded ethanol washes. Antigen retrieval was performed by incubation in 10 mM sodium citrate under high pressure for 5 min, and peroxidases were blocked in 3% hydrogen peroxide for 6 min. Blocking was performed in 10% horse serum for 1 h at room temperature, and samples were incubated with primary antibodies overnight at 4 °C (Supplementary Table 1). Biotinylated secondary antibodies were applied for 1 h at room temperature, and samples were subsequently incubated with avidin-biotin peroxidase using the ABC method (Vector Laboratories). Finally, peroxidase was visualized with the chromogen DAB (3,3’-diaminobenzidine; Vector Laboratories), samples were dehydrated, and coverslips were applied with Permount (VWR) mounting medium. To quantify signal intensity, stained slides were scanned using an Aperio CS2 slide scanner (Leica Biosystems) at 20± magnification. This allows for high resolution access to the entire tissue section. Five square annotation regions (8 × 8 mm) were drawn on each stained tissue section, and the signal intensity within each region was quantified using Aperio algorithms. For experiments where background staining differed between groups, two blinded reviewers counted the number of positive nuclei in a minimum of 10 images per slide. Counts were averaged between the two reviewers and compared between groups. For epithelial and fat pad occupancy comparisons, whole slide scan images were obtained, and ImageJ (NIH) was used to segment space occupied by epithelial cells (colored space) or fat (white space). Percentages were calculated by dividing the area occupied by the entire area analyzed. One entire slide from each mouse in the corresponding lactation studies was included in this analysis, and the mean ±SD for these slides is presented. Publication quality images were collected on a Zeiss Axio Imager.Z1 with 10, 40, or 63× plan-apochromat objectives.

### Cell culture

HC11 cells were purchased from the American Type Culture Collection (ATCC) and were maintained and differentiated as previously described [10, 36, 38]. Briefly, HC11 cells were grown in RPMI (Life Technologies) supplemented with 10% fetal bovine serum (Atlanta Biologicals), 50 μg/mL gentamycin (Life Technologies), 5 μg/mL bovine insulin (MilliporeSigma), and 10 ng/mL recombinant mouse EGF (Life Technologies). For differentiation assays, HC11 cells were grown to confluence, and growth medium was replaced with priming medium (RPMI supplemented with 10% donor horse serum, 50 μg/mL gentamycin, 5 μg/mL bovine insulin, and 1 μg/mL hydrocortisone [MilliporeSigma]). After 24 h, priming medium was replaced with differentiation medium, which consisted of priming medium plus 1 μg/mL ovine prolactin (National Hormone and Peptide Program). Differentiation timepoints were measured from this point on. CIT3 cells were a generous gift from Dr. Peggy Neville and were maintained in DMEM/F12 (Life Technologies) supplemented with 2% FBS, 1% penicillin-streptomycin, 10 μg/mL bovine insulin, and 5 ng/mL recombinant mouse EGF. CIT3 cells were differentiated in the same manner as HC11 cells, with the exception of the media formulations. CIT3 priming media contained DMEM/F12 supplemented with 2% FBS, 1% penicillin-streptomycin, 10 μg/mL bovine insulin, and 3 μg/mL hydrocortisone, and CIT3 differentiation media contained the CIT3 priming media plus 3 μg/mL ovine prolactin. SUM159 cells were purchased from the ATCC and were maintained in DMEM containing 10% fetal bovine serum and 1% penicillin-streptomycin [21, 54, 55, 63]. *Sim2s* overexpressing cell lines have been previously described and are tagged with a FLAG (DYKDDDDK) epitope [10, 21]. Stable *shSim2* cell lines have also been previously validated and described [10, 21]. Mutant *Sim2s* constructs were designed in-house and generated by Life Technologies. Briefly, for *Sim2s-pATMΔ* cells, the serine at positions 35, 115, and 363 was mutated to alanine, as previously described [21]. For *Sim2s-LIRΔ* cells, the consensus LIRs at positions 95 and 193 were mutated from FVFV and YLKI to AVFA and ALKA, respectively [64]. The vector used to transiently express EGFP in our study, pRP[Exp]-CMV.hSIM2[NM_009586.5]/EGFP, was constructed and packaged by Vectorbuilder (vector ID: VB211123-1158drr).

### Isolation of mitochondrial, cytoplasmic, and nuclear fractions

Crude mitochondrial fractions were extracted by differential centrifugation, as previously described [65]. Briefly, a large cell pellet was lysed on ice in RSB hypo buffer (10 mM NaCl, 1.5 mM MgCl_2_, 10 mM Tris-HCl, pH 7.5). Cell lysis was aided by dounce homogenization, and lysis was halted by addition of MSH buffer (210 mM mannitol, 70 mM sucrose, 5 mM Tris-HCl, 1 mM EDTA, pH 7.5). All centrifugations were performed in a prechilled (4 °C) centrifuge. Cell suspensions were centrifuged at 600 × *g* for 5 min, and the supernatant was collected in a new tube. This step was repeated, and the cell pellets containing unbroken cells and nuclei were discarded each time or saved for nuclei isolation. Supernatants were then centrifuged at 7000 × *g* for 10 min, and the pellet was retained. Supernatants from this step were concentrated using Amicon Ultra centrifugal filters (Sigma Aldrich) to generate cytoplasmic fractions per the manufacturer’s instruction. The cell pellet was resuspended in MSH buffer, and the centrifugation and wash steps were repeated. Finally, the suspension was spun at 10,000 × *g* for 10 min, and the supernatant was discarded. The crude mitochondrial pellet was resuspended in high salt (50 mM HEPES, 500 mM NaCl, 1.5 mM EDTA, 10% glycerol, and 1% Triton X-100 at pH 7.5) or RIPA lysis buffer (Life Technologies) for subsequent protein isolation. Lysis buffers were supplemented with 1 mM Na_3_VO_4_ and 1 mM complete ULTRA tablets mini EDTA-free Easy Pack (Roche) to inhibit phosphatase and protease activity, respectively. For digestion of OMM proteins, crude mitochondrial pellets were resuspended in isotonic buffer (300 mM sucrose, 10 mM Hepes, pH 7.4) and divided into two fractions. One fraction was subjected to proteinase K (0.2 mg/ml, Thermo Fisher Scientific) digestion for 20 min on ice, and the other fraction was maintained on ice during this time. Both fractions were then centrifuged at 10,000 × *g* for 10 min at 4 °C and resuspended in high salt lysis buffer. Nuclei were purified from discarded cell pellets by resuspension in GRO lysis buffer (2 mM MgCl_2_, 3 mM CaCl_2_, 0.5% NP-40, 10 mM Tris-HCl, pH 7.4) and dounce homogenization. Homogenates were filtered through a 40 μm cell strainer (VWR) and spun at 500 × *g* for 5 min. Nuclear pellets were resuspended in high salt lysis buffer.

### Energy phenotyping

Energy phenotypes were generated on a Seahorse XFe96 Bioanalyzer (Agilent) using the XF Cell Mito Stress Test (Agilent), as previously described [38]. The third basal measurements were used to calculate the mean OCR and ECAR of each group along with their standard deviations. Cell number was normalized by DC protein assay (BioRad) for cell line data or by DNA quantitation (CyQUANT, Thermo Fisher Scientific) for PMECs. Quadrants were defined by the least significant consecutive differences in the means of the control groups for both OCR and ECAR. Data are presented as the mean ± SD for both OCR and ECAR. A minimum of six biological replicates were included in each analysis, and each experiment was repeated a minimum of two times.

### Transmission electron microscopy

TEM samples were fixed in 0.1 M sodium cacodylate buffer (Electron Microscopy Sciences, EMS) containing 2% glutaraldehyde (EMS) and 2.5% formaldehyde (EMS) for 1 h at room temperature followed by overnight fixation at 4 °C. Samples were washed and stored in 0.1 M sodium cacodylate buffer until processing by Dr. H. Ross Payne in the Image Analysis Laboratory at Texas A&M University. We have described the sample processing for TEM previously [38]. Immunogold labeling of SIM2s was also performed by Dr. H. Ross Payne. Briefly, HC11 cells overexpressing *Sim2s* were differentiated for 24 h and fixed in 4% paraformaldehyde and 0.1% glutaraldehyde in PBS. After fixation, samples were enrobed in 1.5% agar. En bloc staining was performed with 0.2% uranyl acetate. Sections were then dehydrated in increasing ethanol concentrations and infiltrated with white resin. Finally, samples were polymerized with fresh LR white resin, and semithin sections were cut for immunogold staining. Sections were blocked with 0.1 M glycine for 15 min and then with 5% BSA and 0.1% cold water fish skin gelatin for 15 min. After blocking, the primary SIM2s antibody (Aviva Systems Biology) was incubated on sections for 16 h at 4 °C, and secondary antibody conjugates were incubated 1.2 h at room temperature. Gold (10 nM) conjugate was diluted 1:40 and incubated on sections. Finally, sections were fixed briefly in 1% glutaraldehyde and post-stained in 2% uranyl acetate and Reynold’s lead citrate. TEM images were collected on a Morgagni 268 (FEI) system. All images were auto-corrected for contrast using identical parameters, and additional image analysis was performed in ImageJ. Mitochondrial lengths were measured in ImageJ by calculating the longest linear distance covered by an individual mitochondrion. To better address mitochondrial fusion in two-dimensional space, we grouped mitochondria into three categories according to length: punctate/round mitochondria (<1 μm), mid-length mitochondria (1–2 μm), and elongated mitochondria (>2 μm). These categories are presented as percentages of the total number of mitochondria analyzed (>200). Autophagic vesicles were quantified from TEM images that contained approximately 8.5 μm^2^ of cell area each.

### RNA isolation and qPCR

RNA isolation, cDNA reverse transcription, and qPCR amplification methods have been thoroughly described in our recent publication [38]. RNA was extracted with the Roche High Pure RNA Isolation kit, cDNA was reverse transcribed with the BioRad iScript cDNA Synthesis kit, and qPCR was conducted with Promega GoTaq qPCR Master Mix on a BioRad CFX384 qPCR instrument. We assessed the expression of *Csn2* and *Opa1* using the house-keeping gene *Actb* for normalization with the following primers: *Csn2* forward 5’-TGTGCTCCAGGCTAAAGTTCACT-3’, *Csn2* reverse 5’-GGTTTGAGCCTGAGCATATGG-3’, *Opa1* forward 5’-TCTTCACTGCAGGTCCCAAAT-3’, *Opa1* reverse 5’-CTGACACCTTCCTGTAATGCTTG-3’, *Actb* forward 5’-GCAACGAGCGGTTCC-3’, and *Actb* reverse 5’-CCCAAGAAGGAAGGCTGGA-3’. The 2^-ΔΔCt^ method was used to calculate relative expression relative to *Actb* and the experimental control for each assay. Data are depicted as the mean fold ± 1 standard deviation, and statistical analysis was performed on the ΔCt values prior to logarithmic transformation.

### Immunoblotting

Proteins were isolated from cultured cells, as previously described [38], in high salt lysis buffer containing 50 mM HEPES, 500 mM NaCl, 1.5 mM EDTA, 10% glycerol, 1% Triton X-100, 1 mM Na_3_VO_4_, and 1 mM complete ULTRA tablets mini EDTA-free Easy Pack at pH 7.5. Protein concentrations were measured by DC protein assay (BioRad), and equivalent amounts of protein were combined with Laemeli buffer, boiled for 5 min, and loaded into SDS-PAGE gels for electrophoresis. Western blotting was performed as previously described [21, 38]. Bands were digitized on a ChemiDoc MP Imaging System (BioRad), and band intensities were measured using ImageJ. Band intensity was normalized to the loading control or unphosphorylated protein to control for protein loading and was subsequently normalized the experimental control. Final band intensities represent a value relative to the experimental control and normalized for protein content. Antibodies, dilutions, and suppliers are described in Supplementary Table 1.

### Co-immunoprecipitation

Interactions between proteins were assessed by co-immunoprecipitation (co-IP) using FLAG-conjugated magnetic beads (MilliporeSigma) in HC11-*Sim2s* cells, which express the peptide tag FLAG. HC11 control cells were used as a negative control, because they do not express the peptide tag and should not interact with FLAG-conjugated beads. Magnetic beads conjugated to mouse-IgG (Cell Signaling Technology) were also used as an additional negative IgG control and should not recognize any specific protein. Cells were lysed in RIPA buffer, flash frozen, thawed, and spun at 14,000 × *g* for 30 min to extract proteins. Lysates were used immediately for Co-IP experiments to avoid additional freeze-thaw cycles. Freshly isolated lysates were incubated overnight with FLAG-conjugated beads. Prior to incubation with lysates, FLAG-conjugated beads were blocked overnight with 5% BSA (Thermo Fisher Scientific). Protein-bead complexes were washed carefully five times in TBS, and bound proteins were eluted from beads in SDS buffer (125 mM Tris-HCl, pH 6.8, 4% SDS, 20% (v/v) glycerol, and 0.004% bromophenol blue). Each sample was supplemented with 2-mercaptoethanol, boiled for 5 min, and western blotted as previously described [21, 38]. Antibodies, dilutions, and suppliers are described in Supplementary Table 1.

### Proximity ligation assay

Interactions were further analyzed by proximity ligation assay (PLA) using MilliporeSigma Duolink In Situ PLA technology following the manufacturer’s protocol. SUM159 pLPCX *SIM2s-FLAG* cells were plated on glass coverslips and cultured to approximately 80% confluence. To begin the experiment, cells were fixed in 2% paraformaldehyde (Santa Cruz) for 15 min and then permeabilized with 0.1% Triton X-100 in PBS for 15 min. Cells were blocked using the Duolink Blocking Solution for 60 min at 37 °C and then incubated in primary antibody (Supplementary Table 1) for 3 h at room temperature. Samples where no primary antibody was added were included as negative controls. After primary antibody incubation, Duolink PLA probes, anti-mouse PLUS (DUO92001) and anti-rabbit MINUS (DUO92005), were added to the cells and incubated at 37 °C for 1 h. Signal was generated using Duolink In Situ Detection Reagents Orange (DUO92007) following the manufacturer’s protocol of ligation for 30 min at 37 °C and amplification for 100 min at 37 °C. Following signal amplification, cells were either stained with 16 mM Hoechst (Thermo Fisher Scientific) and mounted on glass microscope slides using Prolong Anti-fade Gold (Thermo Fisher Scientific) or the cover slips were mounted on glass microscope slides using Duolink In Situ Mounting Media with DAPI. Images were taken on a Zeiss Axio Imager.Z1 with 40× (quantification) and 63× (representative images) plan-apochromat objectives. Positive puncta were quantified per cell from 3–6 images per cell line and antibody.

### MitoTimer, MitoTracker, and LysoTracker

Fluorescent reporter assays were performed on live HC11 and CIT3 cells at the Image Analysis Laboratory at Texas A&M University on a Zeiss LSM 780 NLO Multiphoton microscope with a 63× plan apochromat objective. The *pMitoTimer* construct (Addgene, 52659, deposited by the Zhen Yan Laboratory) was transiently transfected in HC11 control and *Sim2s* cells, as previously described [38]. Fluorescent images were collected beginning at 24 h of priming and proceeding through 48 h of differentiation. For image analysis, we excluded saturated pixels (gray level = 225) and measured single channel mean fluorescent intensities (MFI). The MFI for RFP was divided by the MFI for GFP to generate a red:green ratio for each image. A minimum of 10 images were evaluated per sample. Average ratios were then compared between groups. MitoTracker, LysoTracker, and Mitotracker DeepRed (Life Technologies) staining was performed for 30 min at 37 °C in phenol red-free media. After dye incubation, cells were stained with 16 mM Hoechst (Thermo Fisher Scientific), washed with PBS, and a minimum of 10 images were collected for each sample. Image analysis was performed with ImageJ, and saturated pixels were excluded. MFI of each dye was calculated and compared between groups.

### Statistical analysis

At a minimum, all experiments were performed with biological triplicates and technical duplicates. Each comparison was confirmed in a minimum of two independent experiments. Box and whisker plots encompass the 25th to 75th percentile, and the whiskers extend to the minimum and maximum values. The central line indicates the mean of each group. Wherever error is indicated, standard deviation has been used. Statistical analyses were performed in JMP Pro or GraphPad Prism. Significant differences between continuous variables were assessed using two-sided student’s *t*-tests, and *p* < 0.05 was considered statistically significant. Normal distribution was confirmed prior to utilization of student’s *t*-tests. The data that support the findings of this study are available from the corresponding author upon request.

## Supplementary information

original data file

Supplemental Table 1

Supplemental Figure 1

Supplemental Figure 2

Supplemental Figure 3

Supplemental Figure 4

Supplemental Figure 5

Reproducibility_checklist
