# Supplementary material for: SIM2s directed Parkin-mediated mitophagy promotes mammary epithelial cell differentiation
Source: Cell Death Differ. 2023 Mar 25;30(6):1472–87. doi: 10.1038/s41418-023-01146-9 (PMC10244402; doi:10.1038/s41418-023-01146-9)
Supplement: Supplementary file 1 — original data file [file 41418_2023_1146_MOESM1_ESM.pdf]

SIM2s directed Parkin-mediated mitophagy promotes the function and survival of mammary epithelial cells

Lilia Sanchez, Jessica Epps, Steven Wall, Cole McQueen, Scott J. Pearson, Kelly Scribner, Elizabeth A. Wellberg, Erin D. Giles, Monique Rijnkels*, Weston W. Porter*

Supp. Fig. 6. In this supplemental materials section, we provide original western blot files for the proteins we probed for in our manuscript. Molecular weight is marked on the final figures.

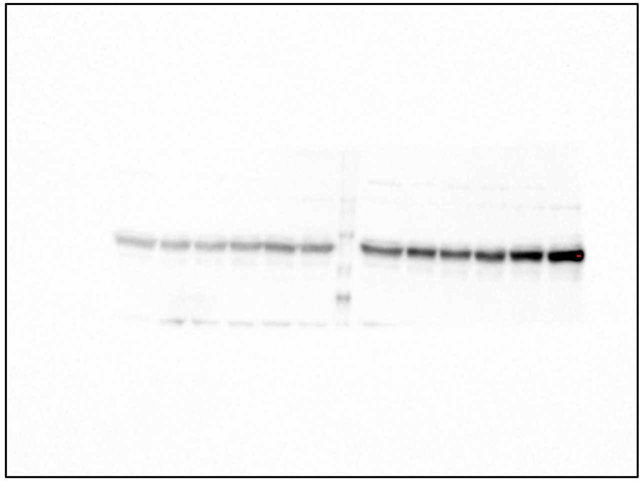

TOMM70

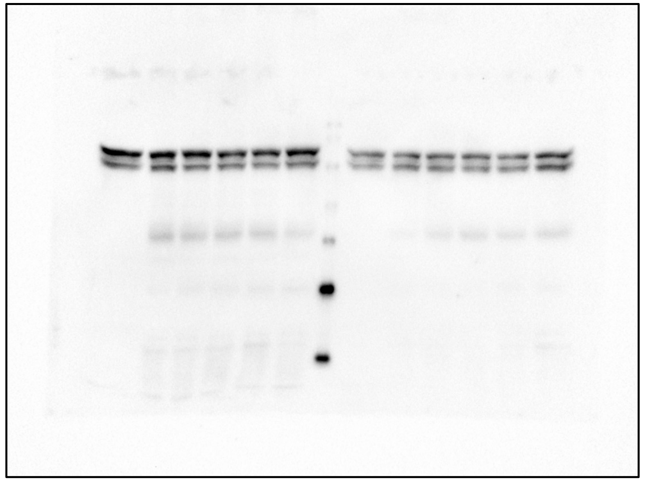

OPA1

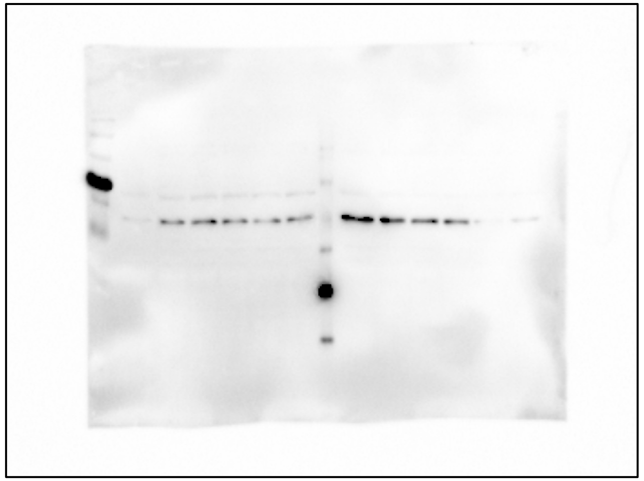

DRP1

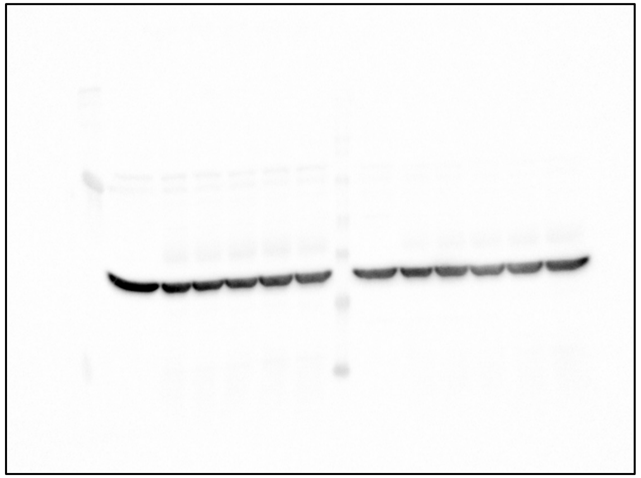

ACTB

Original blots in Fig. 3 Original blots showing TOMM70, OPA1, DRP1, and ACTB for representative blots in figure 3 in manuscript.

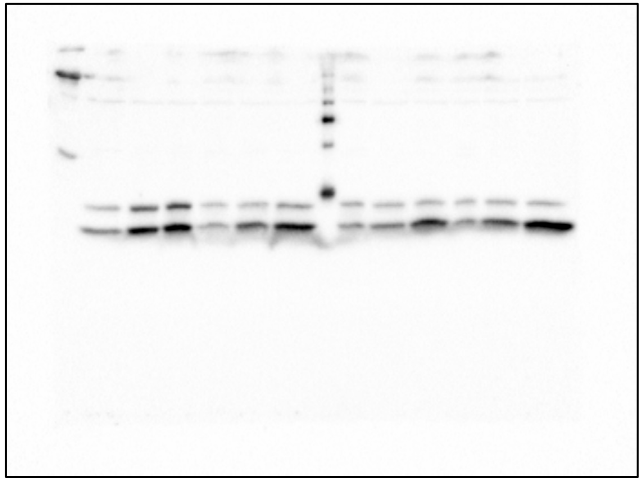

LC3B

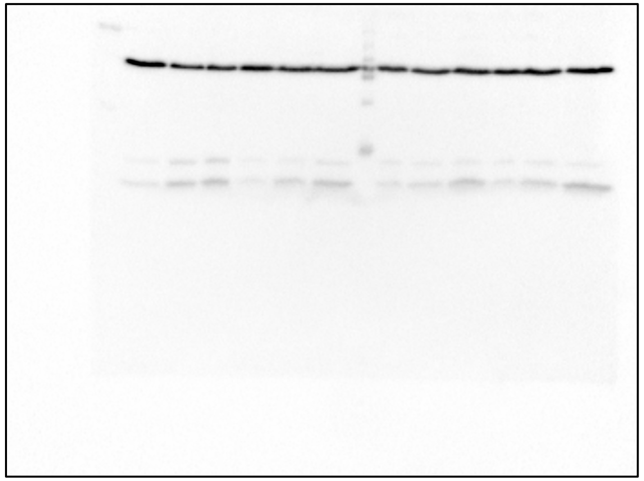

ACTB

Original blots in Fig. 4 Original blots showing LC3B and ACTB for representative blots in figure 4 in manuscript.

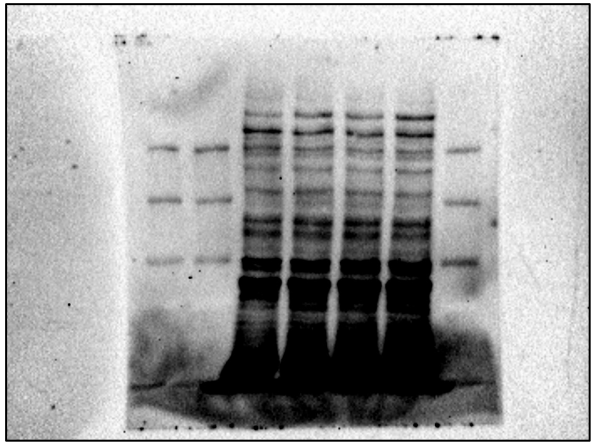

pATM

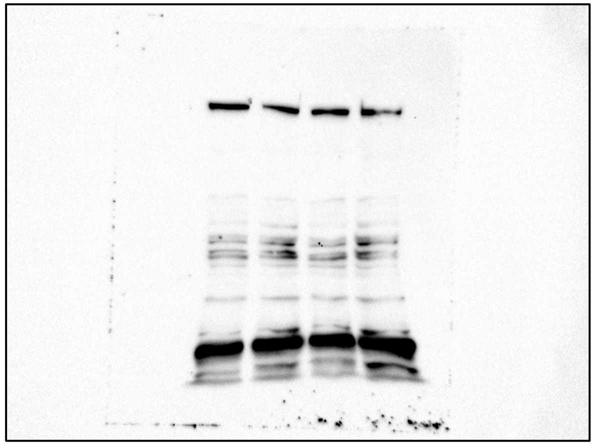

ATM

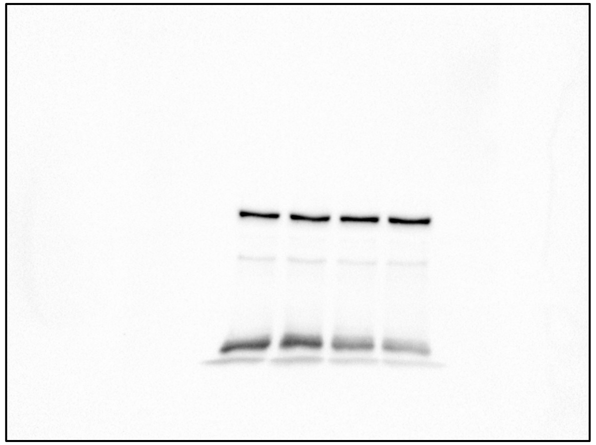

VCL

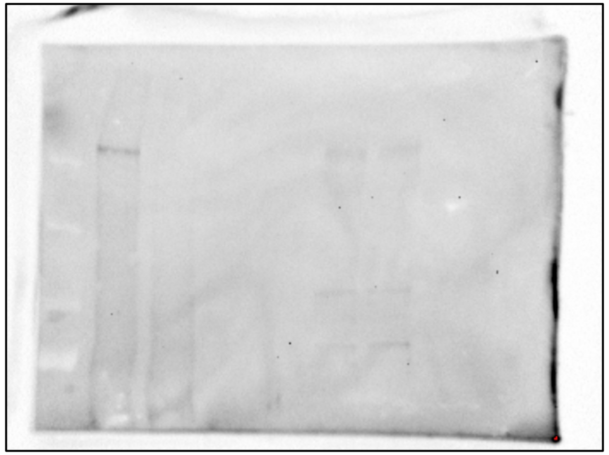

ATM, IgG, 10%iniput

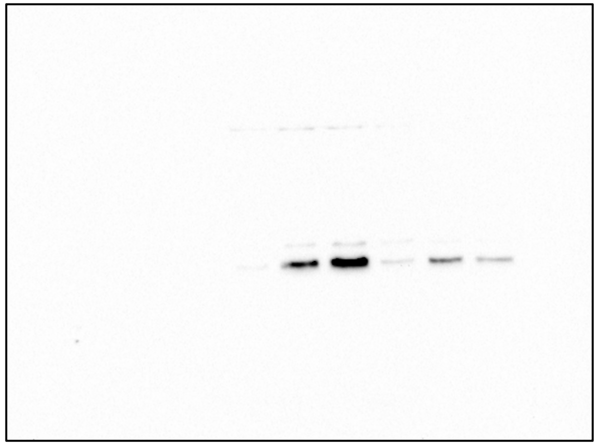

LC3B

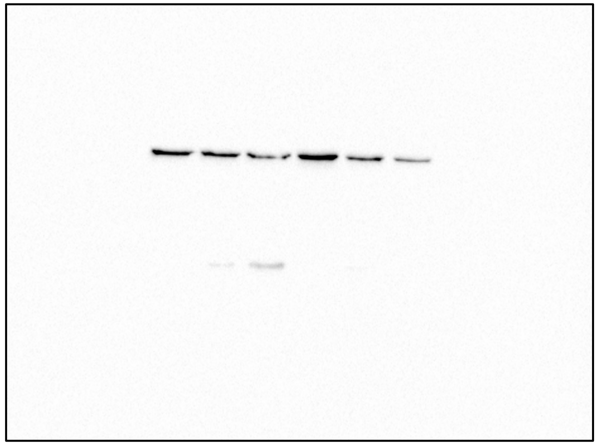

ACTIN

Original blots in Fig. 5 Original blots showing pATM, ATM, and VCL for representative blots in figure 4 in manuscript.

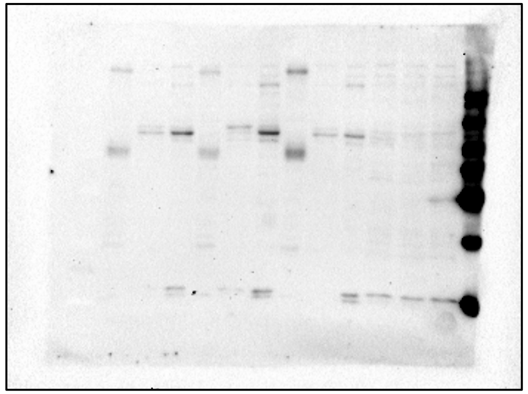

SIM2

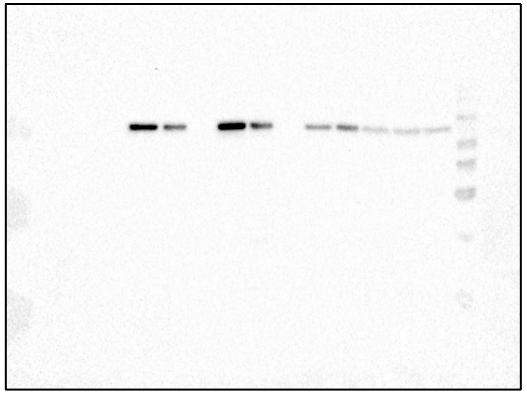

TOMM70

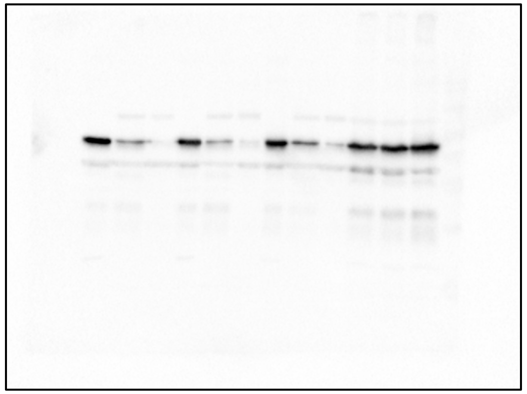

TUBA

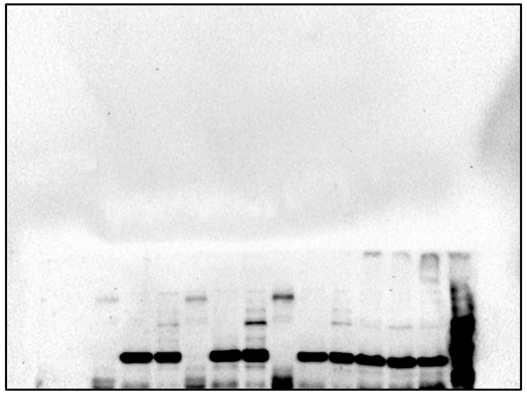

PARP1

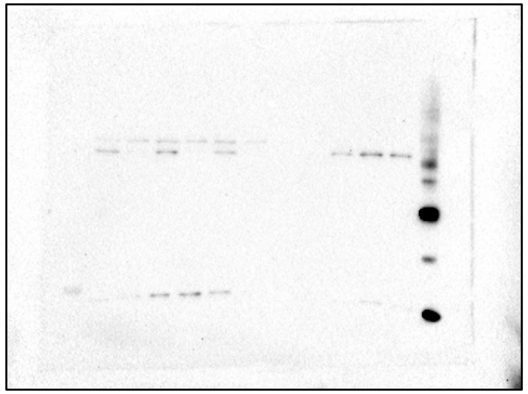

SIM2

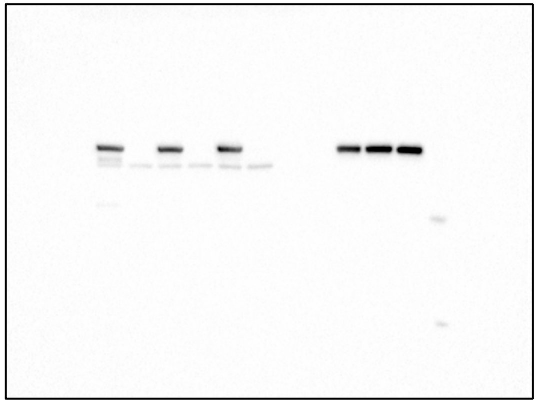

TOMM70

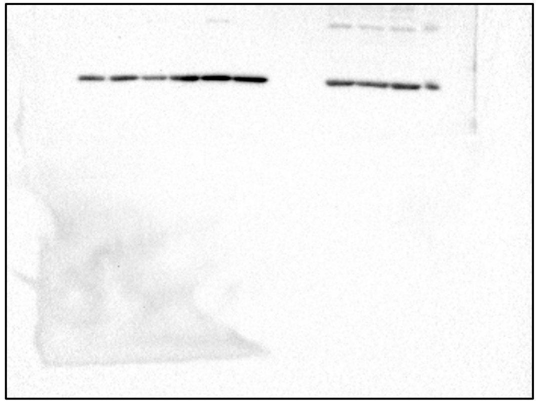

COX4

Original blots in Fig. 6 Original blots showing SIM2, TOMM70, TUBA, PARP1, and COX4 for representative blots in figure 6 in manuscript.

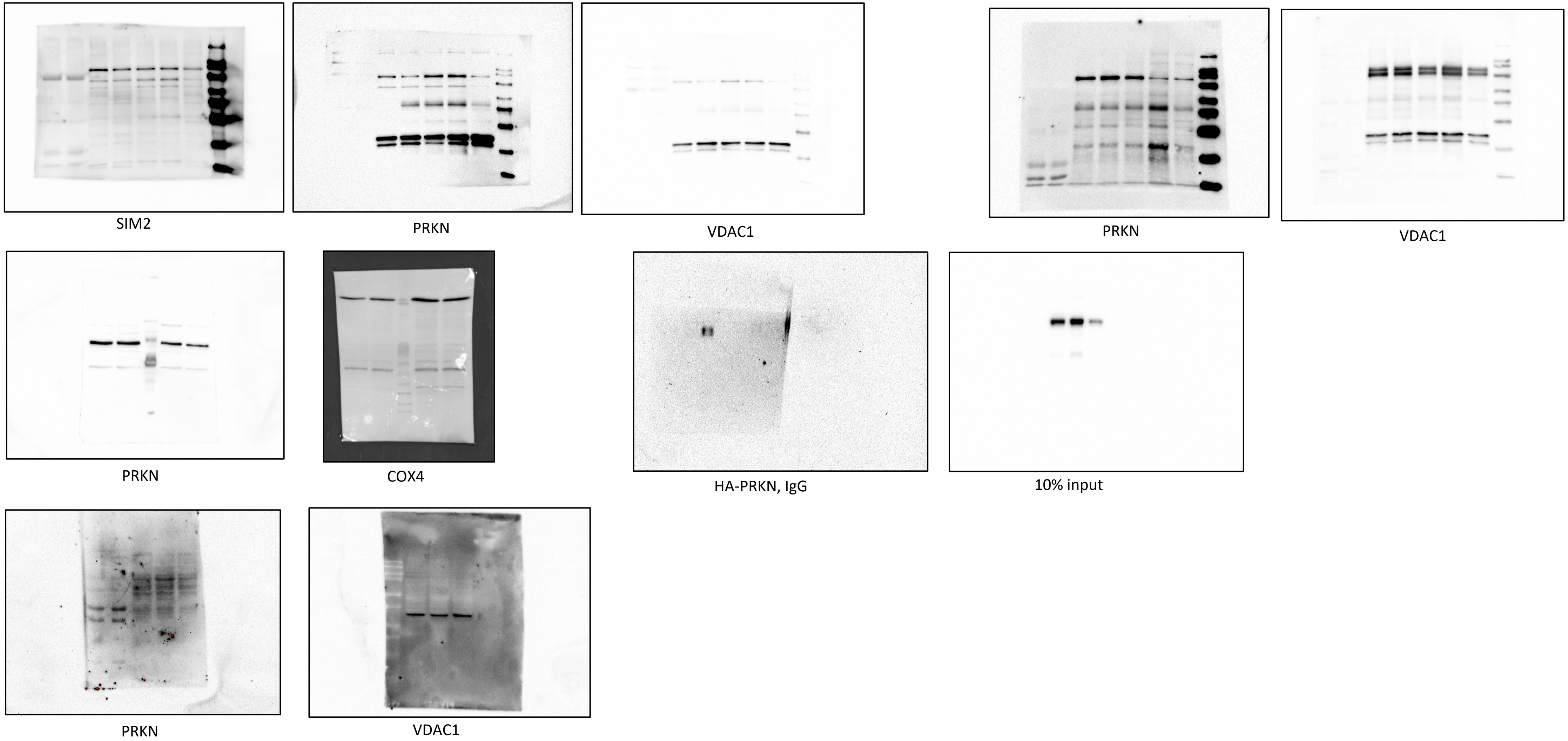

Original blots in Fig. 7 Original blots showing SIM2, TOMM70, TUBA, PARP1, COX4, HA-PRKN, LC3B, ACTIN for representative blots in figure 6 in manuscript.

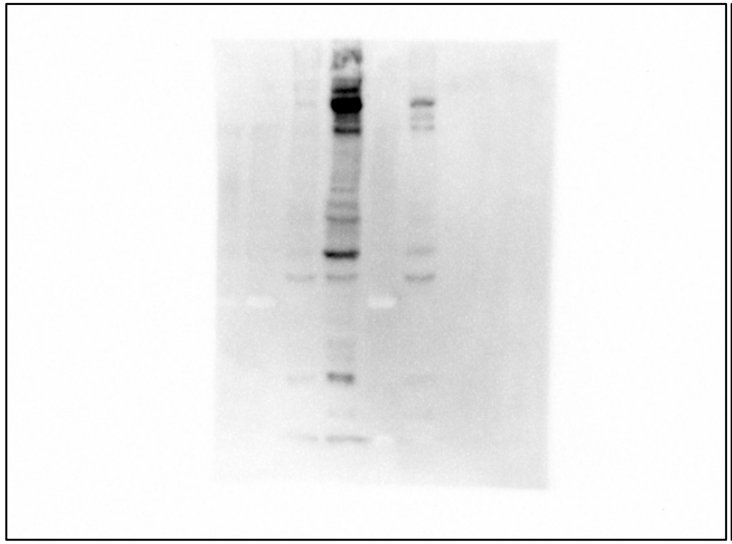

pATM

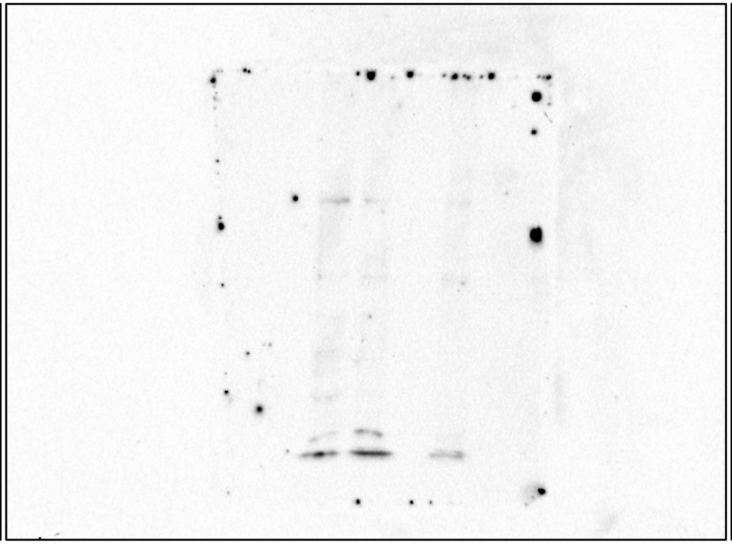

ATM

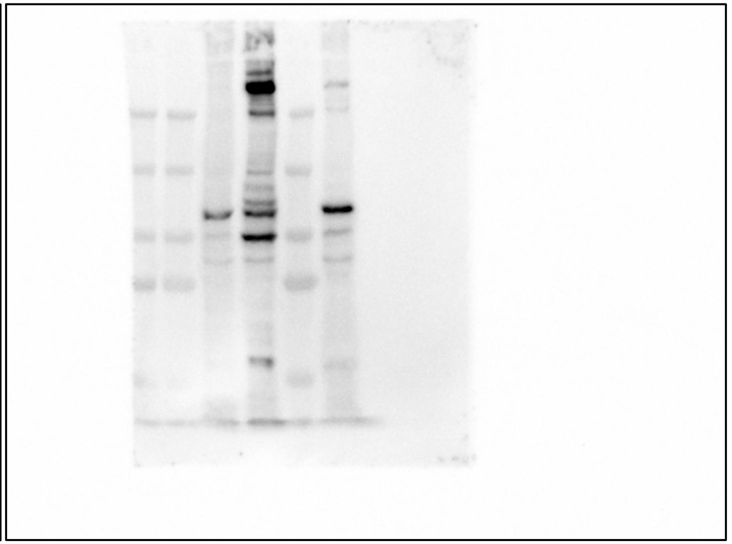

VCL

Original blots in Supp. Fig 5. Original blots showing pATM, ATM, and VCL in supplemental figure 5.
