# Supplementary material for: SIM2s directed Parkin-mediated mitophagy promotes mammary epithelial cell differentiation
Source: Cell Death Differ. 2023 Mar 25;30(6):1472–87. doi: 10.1038/s41418-023-01146-9 (PMC10244402; doi:10.1038/s41418-023-01146-9)
Supplement: Supplementary file 2 — Supplemental Table 1 [file 41418_2023_1146_MOESM2_ESM.docx]

**Supplemental Table 1**

| **Antibody** | **Dilution** | **Vendor** | **Catalog Number** |
| --- | --- | --- | --- |
| Anti 8-OHdG monoclonal antibody | IHC 1:100 | Genox Corporation | MOG-020P |
| ACTB | WB: 1:5000 | Cell Signaling Technology | 37005 |
| ATM | WB: 1:500 | Millipore | 07-1286 |
| CCasp-3 | IHC: 1:100 | Cell Signaling | 9661 |
| COX4 | WB: 1:1000 IHC: 1:500 | Thermo Scientific | PA5-19471 |
| CSN2 | WB: 1:250 IHC: 1:100 | Santa Cruz Biotechnology | sc-166530 |
| DRP1/ DNM1L | WB: 1:1000 IHC: 1:100 | EMD Millipore | ABT 155 |
| FLAG | WB: 1:1000 | Cell Signaling Technology | 8146S |
| HA |  |  |  |
| LC3B | WB: 1:1000 | Novus Biologicals | NB100-2220 |
| MKI67 |  |  |  |
| OPA1 | WB: 1:1,000 IF: 1:100 | BD Biosciences | 612607 |
| p-STAT3 (Tyr705) | WB: 1:1000 IHC: 1:250 | Cell Signaling Technology | 9131 |
| PARP1 | WB: 1:1000 | Cell Signaling Technology | 9542 |
| pATM (Ser-1981) | WB: 1:500 | ECM Biosciences | AM3661 |
| p-STAT5 | IHC: 1:100 | Cell Signaling | 9358S |
| PPARGC1A |  |  |  |
| PRKN | WB: 1:500 IHC: 1:200 | Abcam | ab77924 |
| SIM2 | WB: 1:500 | Aviva | AB81292 |
| SLC34A2 |  |  |  |
| SQSMT1 | IHC: 1:500 | MBL | PM045 |
| TOMM70 | WB: 1:1000 IHC: 1:100 | Proteintech | 14528-1-AP |
| TUBA | WB: 1:1000 | Santa Cruz Biotechnology | sc-8035 |
| VCL | WB: 1:1000 | BD Biosciences | MAB6896 |
| VDAC1 | WB: 1:1000 | Abcam | ab14734 |
| Anti-mouse HRP | WB: 1:5000 | Cell Signaling Technology | 7076 |
| Anti-rabbit HRP | WB: 1:5000 | Cell Signaling Technology | 7074 |
| Anti-mouse biotinylated | IHC: 1:250 | Vector Laboratories | BMK-2202 |
| Anti-rabbit biotinylated | IHC: 1:250 | Vector Laboratories | BA-1000 |
