# Supplementary figures and images for: SIM2s directed Parkin-mediated mitophagy promotes mammary epithelial cell differentiation
Source: Cell Death Differ. 2023 Mar 25;30(6):1472–87. doi: 10.1038/s41418-023-01146-9 (PMC10244402; doi:10.1038/s41418-023-01146-9)

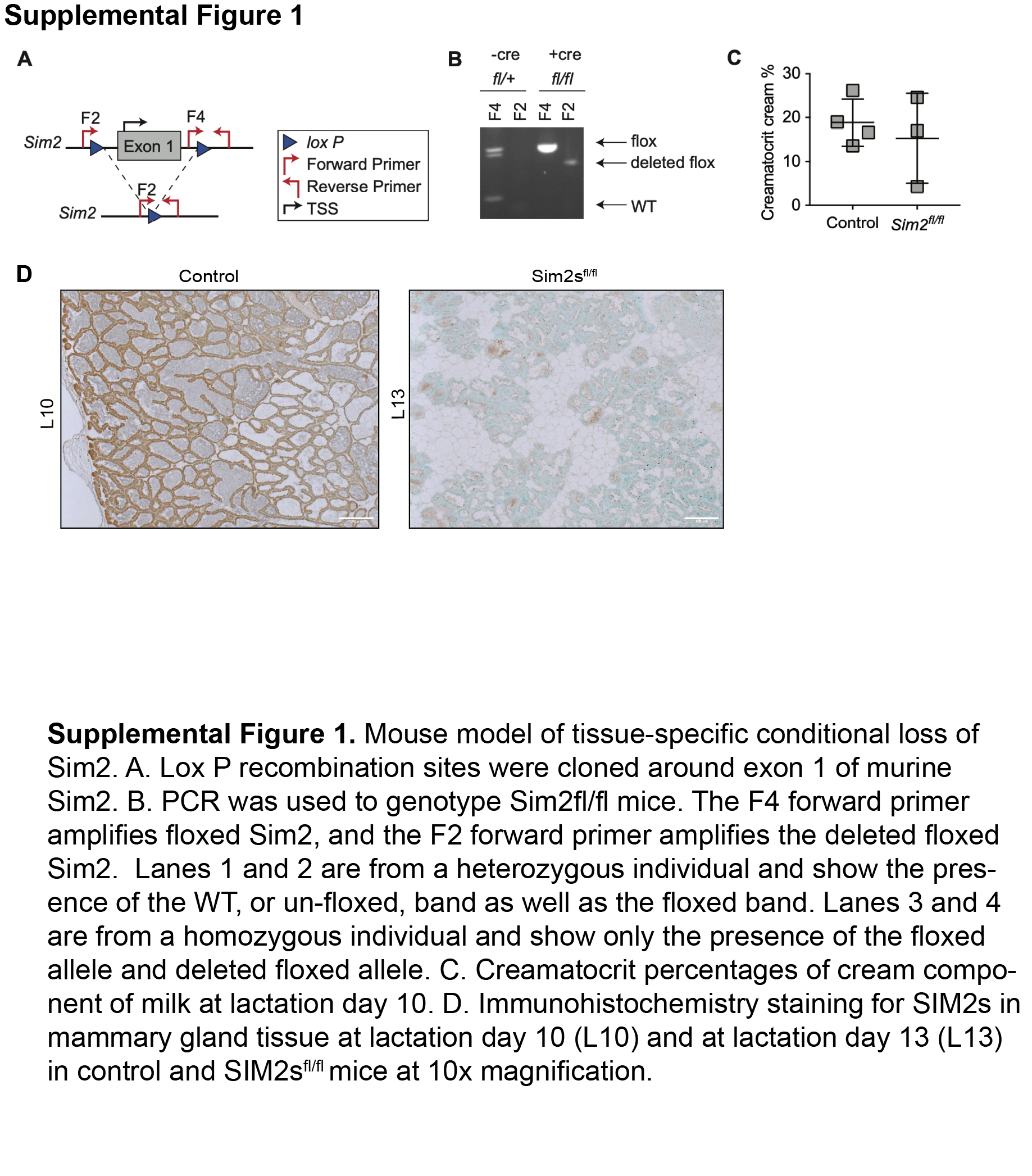

Supplement: Supplementary file 3 — Supplemental Figure 1 [file 41418_2023_1146_MOESM3_ESM.png]

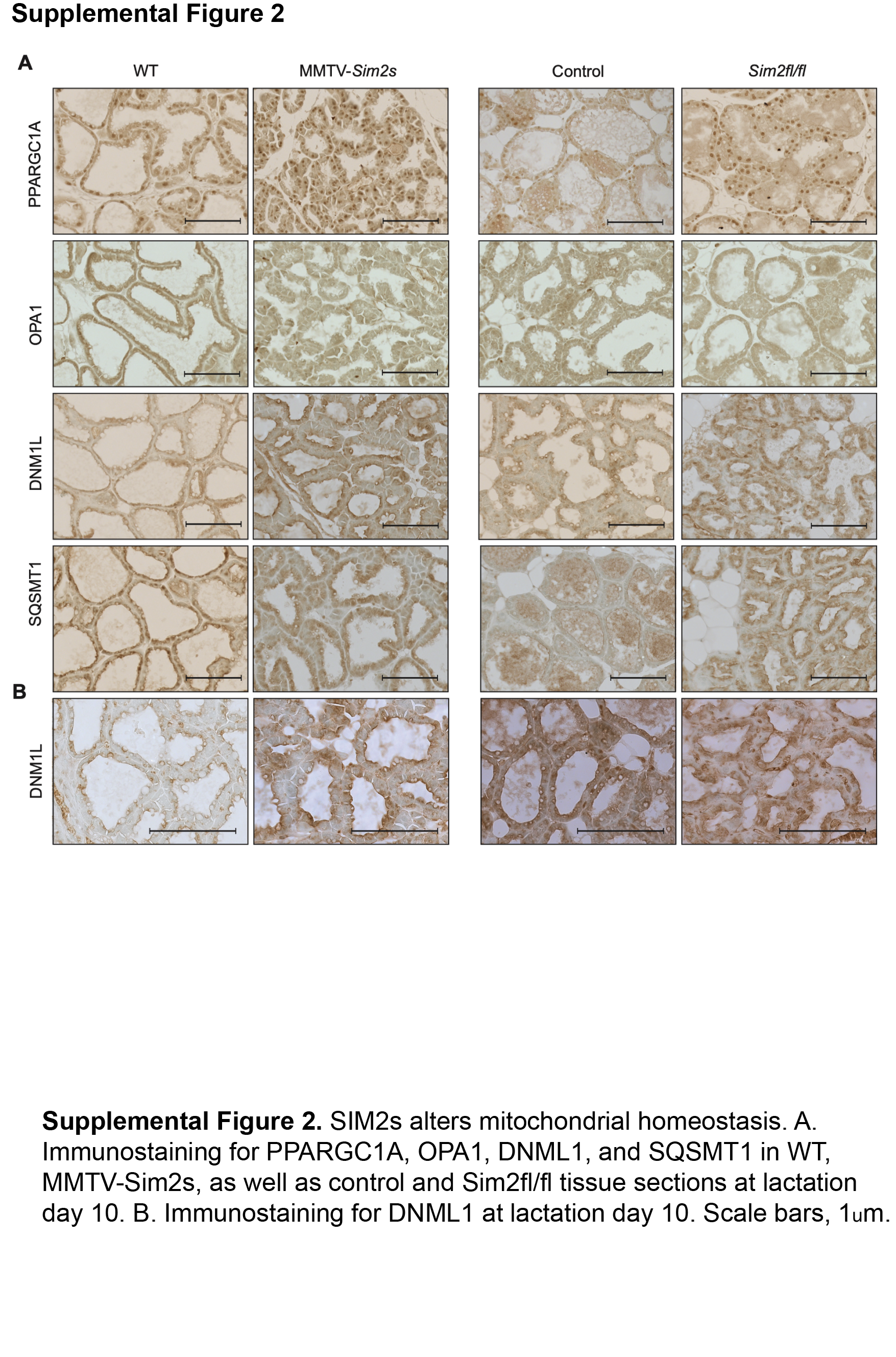

Supplement: Supplementary file 4 — Supplemental Figure 2 [file 41418_2023_1146_MOESM4_ESM.png]

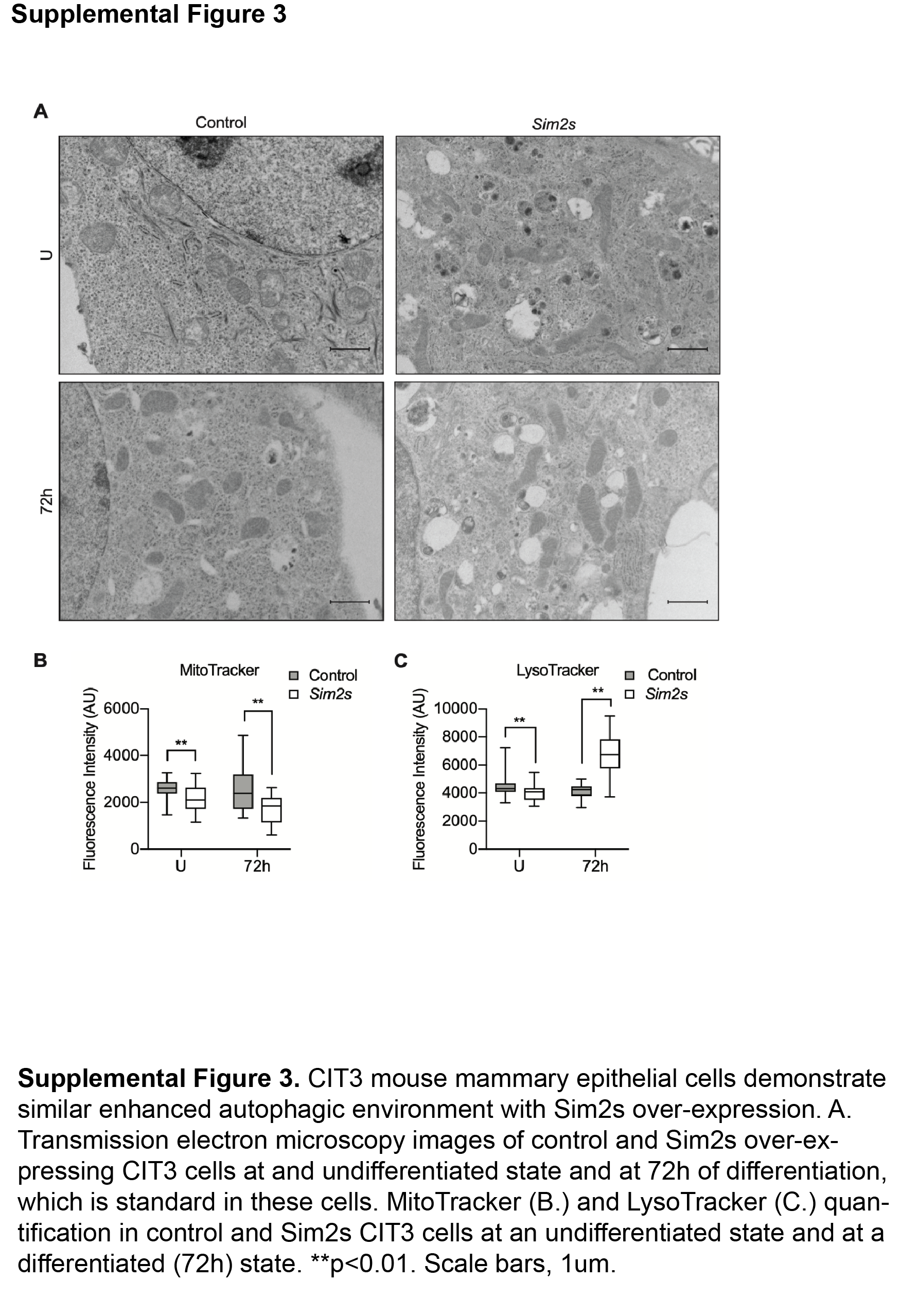

Supplement: Supplementary file 5 — Supplemental Figure 3 [file 41418_2023_1146_MOESM5_ESM.png]

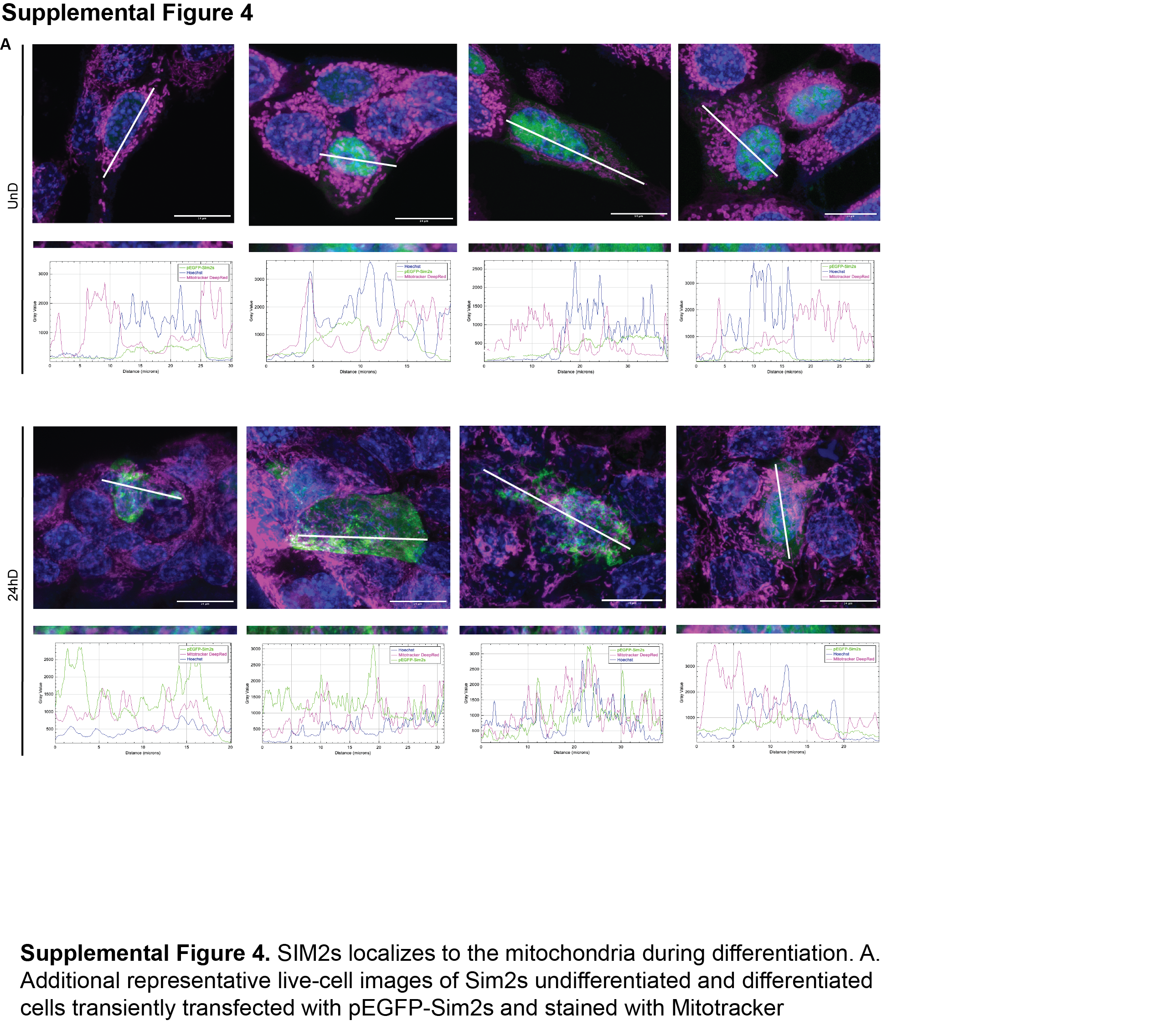

Supplement: Supplementary file 6 — Supplemental Figure 4 [file 41418_2023_1146_MOESM6_ESM.png]

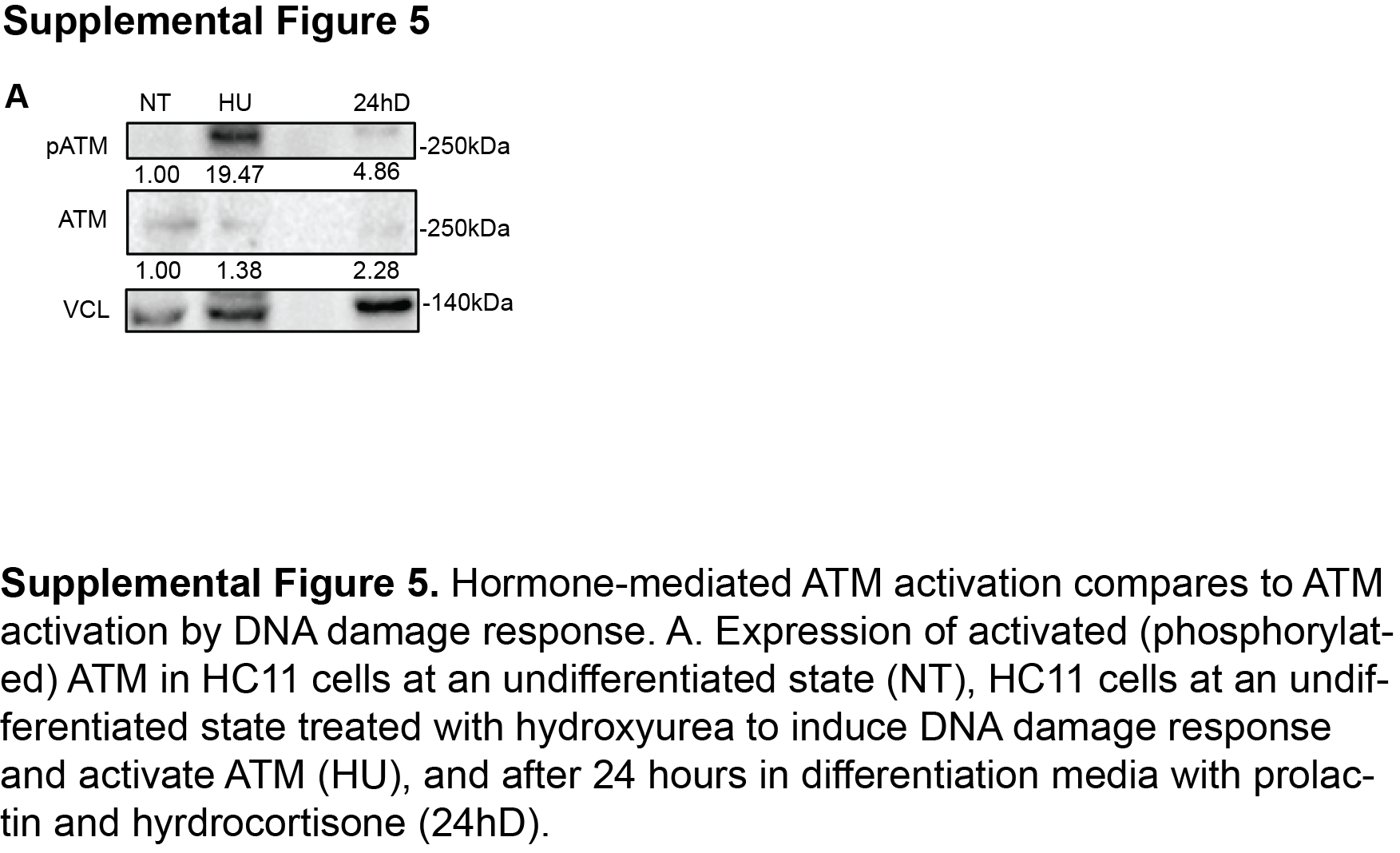

Supplement: Supplementary file 7 — Supplemental Figure 5 [file 41418_2023_1146_MOESM7_ESM.png]
